# Effects of AMF on tobacco growth in continuous cropping soils: impacts on soil chemical properties and rhizosphere microbial diversity

**DOI:** 10.3389/fpls.2026.1827990

**Published:** 2026-07-08

**Authors:** Jiangyuan Wang, Junying Li, Xingyan Fan, Xinchun Niu, Xiaopeng Deng, Fuzhao Nian, Han Wang, Ningbo Han, Shanqin Yang, Jiabin Dong, Lili Tang, Quanru Shi, Yating Liu, Di Liu

**Affiliations:** 1 College of Tobacco Science, Yunnan Agricultural University, Kunming, China; 2 Yunnan Academy of Tobacco Agricultural Sciences, Kunming, China; 3 Weishan County Branch of Dali Tobacco Company, Dali, China; 4 Zhongke Biotechnology (Yunnan) Co., Ltd., Jianshui, China; 5 Sichuan Tobacco Company, Panzhihua Branch, Panzhihua, China; 6 Luzhou Branch of Sichuan Provincial Tobacco Company, Luzhou, China

**Keywords:** allelochemicals, arbuscular mycorrhizal fungi, continuous cropping obstacle, rhizosphere microbiome, soil enzymes, tobacco

## Abstract

**Introduction:**

Continuous tobacco cropping leads to reduced nutrient bioavailability, severe autotoxicity, and disrupts the rhizosphere microbial balance, ultimately reducing leaf yield and quality. Inoculation with arbuscular mycorrhizal fungi (AMF) can promote plant growth by enhancing nutrient uptake and modulating soil microbial communities.

**Methods:**

This study investigated the mechanisms underlying growth inhibition in consecutively cropped soils and evaluated the potential of AMF to alleviate such cropping stress through a pot experiment.

**Results:**

Compared with non-inoculated plants, AMF inoculation significantly improved photosynthetic parameters, agronomic traits, and biomass during the vigorous growth stage. It also increased antioxidant enzyme activities in both leaves and roots, elevated soil enzyme activities (catalase, sucrase, polyphenol oxidase), and enhanced soil nitrogen, phosphorus, and potassium content. Furthermore, AMF inoculation enriched soil microbial diversity, particularly increasing the abundance of *Lysobacter*, and exerted stronger effects on the fungal community than bacteria. AMF inoculation also reduced concentrations of allelopathic compounds in soil, including hydroxybenzoic acid, vanillic acid, p-coumaric acid, ferulic acid, and myristic acid. In contrast, tobacco grown in consecutively cropped soils exhibited decreased photosynthetic performance, root growth, biomass, and reduced enzyme activities, including CAT, PAL and SOD in leaves and SOD, CAT, PAL and POD in roots.

**Discussion:**

Overall, continuous cropping negatively affects tobacco growth and soil homeostasis, whereas AMF inoculation promotes plant growth, mitigates allelopathic stressors associated with continuous cropping, and significantly improves soil chemical properties and microbial abundance and functionality.

## 1 Introduction

Continuous cropping, the practice of cultivating the same crop species or family in the same field for two or more consecutive seasons, often suppresses seed germination and reduces crop growth performance, ultimately compromising both yield and quality (Wang et al., 2013; Xi et al., 2019; Chen et al., 2020; Wang et al., 2020). Tobacco (*Nicotiana tabacum* L.) is particularly susceptible to these adverse effects, with continuous cropping leading to significant soil degradation and productivity losses (Bai et al., 2019).

Arbuscular mycorrhizal fungi (AMF) are among the most widespread symbiotic fungi in terrestrial ecosystems, forming mutualistic associations with approximately 80% of terrestrial plants (Yi et al., 2023). AMF enhance host nutrient acquisition and utilization by extending extraradical hyphae that transport essential mineral elements (Chen et al., 2019). Beyond nutrient uptake, AMF play a pivotal role in regulating soil microbial community composition and function. For example, Xie (Xie et al., 2022) showed that AMF interact with soil microorganisms, to facilitate organic matter decomposition and nutrient cycling, processes fundamental to soil ecosystem stability. AMF also contribute to soil structure stabilization by forming hyphal networks that bind soil particles (Zeng et al., 2023). Furthermore Li (Li et al., 2022) reported that AMF extraradical hyphae secrete allelopathic substances, including organic acids and polyamines, which enhance soil particle aggregation. Through altering host root morphology, forming continuous mycelial networks, and releasing root exudates such as organic acids, phosphatases, and protons, AMF modify soil physicochemical properties and create a more favorable rhizosphere environment (Bagyaraj et al., 2015).

In agricultural systems, continuous tobacco cropping significantly degrades soil quality and restricts plant growth. Harnessing the physiological and ecological benefits of AMF offers a promising strategy to mitigate these negative effects.

However, most existing studies primarily focus on the generalized growth-promoting effects exhibited by arbuscular mycorrhizal fungi (AMF) under conditions of nutrient deficiency or salt stress. There remains a lack of in-depth understanding regarding how AMF mitigate the complex issue of “continuous cropping obstacles” in tobacco cultivation—particularly concerning the degradation of tobacco allelochemicals and the restructuring of rhizosphere microbial co-occurrence networks. Although it is known that AMF can alter the composition of root exudates, their specific capacity to reduce autotoxic phenolic acids within high-intensity continuous cropping systems for tobacco has yet to be systematically and rigorously elucidated.

To address this research gap, this study tests the following hypotheses regarding the role of AMF in alleviating continuous cropping stress in tobacco: (1) AMF inoculation will improve tobacco growth performance, photosynthetic efficiency, and biomass accumulation under continuous cropping conditions; (2) AMF will enhance soil nutrient availability (N, P, K) and increase the activities of key soil enzymes; (3) AMF inoculation will reshape the soil microbial community structure, increase microbial diversity, and specifically promote beneficial taxa such as *Lysobacter*; (4) AMF will reduce the accumulation of autotoxic allelopathic compounds in the soil, thereby mitigating their inhibitory effects on tobacco growth. The findings aim to provide a scientific basis and technical support for the application of mycorrhizal technology to enhance crop productivity and promote soil sustainability in continuous cropping systems.

## 2 Materials and methods

### 2.1 Experimental time, location, and materials

A pot experiment was conducted from June to July 2024 in a controlled-environment intelligent greenhouse at Yunnan Agricultural University, Kunming, Yunnan Province, China. The experimental red soil was collected from the top layer (0–20 cm depth) of agricultural fields in Luoping County, Qujing City, Yunnan Province. Two soil types were selected either under continuous tobacco cultivation for five years (designated as C+), or soil from an adjacent field where maize had been conventionally cultivated without tobacco over the same period (designated as C-). After collection, large debris and stones were removed. Importantly, the field-collected soils were deliberately not sterilized. This was strictly necessary to preserve the indigenous microbial communities and allelochemical profiles, thereby allowing us to accurately simulate realistic field continuous-cropping obstacles and evaluate the true ecological remediation effects of the introduced AMF. To standardize the physical matrix conditions (e.g., bulk density, porosity, and water-holding capacity) across all treatments, both soil types were uniformly mixed with chemically inert, sterile vermiculite at an identical 3:1 (v/v) ratio. The basic physicochemical properties of these final soil-vermiculite mixtures were quantified prior to the experiment to verify their baseline characteristics, as presented in **Table 1**. Round plastic pots measuring 35 cm in diameter and 25 cm in height served as cultivation containers. Uniform tobacco seedlings (variety K326, provided by Yuxi Zhongyan Seed Co., Ltd.) were raised using the float tray system for 45 days. Morphologically uniform and healthy seedlings were transplanted. A tobacco-specific compound fertilizer (N-P_2_O_5_-K_2_O = 10-10-25) was applied.

**Table 1:** Physicochemical properties of the experimental base soil.

Treatments	PH	OM(g/kg)	AN (mg/kg)	AP (mg/kg)	AK (mg/kg)	TN (g/kg)	TP(g/kg)	TK (g/kg)
C+	6.97 ± 0.04	35.52 ± 1.47*	56.00 ± 7.00	105.96 ± 17.04*	671.47 ± 14.09*	2.5 ± 0.14**	2.18 ± 0.14*	9.3 ± 0.90
C-	7.07 ± 0.12	30.94 ± 0.10	32.67 ± 4.04	77.52 ± 5.11	412.13 ± 68. 30	2.0 ± 0.01	1.91 ± 0.2	11.2 ± 0.80

C+: Soil from a field where tobacco has been continuously cultivated for five years; C-: Soil from a field where tobacco has not been planted for five years. Each treatment had three replicates, and the two sets of data were analyzed using an independent samples T-test. * Indicates a significant difference between treatment groups under independent samples T-test (*p < 0.05, **p < 0.01, ***p< 0.001). OM, Organic Matter; AN, Alkaline Hydrolyzable Nitrogen; AP, Available Phosphorus; AK, Available Potassium; TN, Total Nitrogen; TP, Total Phosphorus; TK, Total Potassium.

The AMF inoculum (*Funneliformis mosseae*) was obtained from the Institute of Plant Nutrition, Resources and Environment, Beijing Academy of Agriculture and Forestry Sciences, with preservation numbers BGC YN05 and 1511C0001BGCAM0013, and contained 2000 spores per 50 g of soil (Zhan et al., 2018). The inoculum was propagated on maize (*Zea mays* L.) grown in a sterile vermiculite: field soil mixture (1:3, v/v) using the “sandwich” method (Fang et al., 2021.). After 60 days, AMF colonization in maize roots exceeded 90% without contamination, the roots and rhizosphere soil were air-dried, ground, and mixed to prepare the inoculum. For the AMF inoculation plants, 200 g of live AMF inoculum was applied directly around the roots using the sandwich method prior to covering with soil. The non-AMF control received an equal weight of sterilized, inactivated inoculum to maintain substrate consistency. This specific strain was selected because it is a globally distributed dominant species and previous studies have confirmed its exceptional efficacy in enhancing the stress tolerance and nutrient uptake capabilities of tobacco crops (Begum et al., 2022).

### 2.2 Pot experiment design and implementation

The experiment followed a completely randomized two-factor design, with soil type and AMF inoculation as the main factors. Soil conditionsincluded continuous tobacco cropping soil (C+) and non-continuous cropping soil (C-). Inoculation treatments included AMF inoculated (M+) and non-inoculated (M-). This established four treatment groups: C+M+, C+M-, C-M+, and C-M-. This experiment employed a completely randomized two-factor design, with each treatment group comprising 10 independent replicates (one plant per pot). In our statistical model, given that only one plant was cultivated per pot, we designated the “pot” as the primary experimental unit; this approach effectively precluded the occurrence of pseudo replication and appropriately addressed potential nested variability. After 45 days of float seedling cultivation in a sterile, soilless commercial substrate, uniformly sized seedlings (which were randomly sampled, stained, and microscopically confirmed to have 0% background AMF colonization prior to the experiment) were transplanted into pots containing 8 kg of the soil-vermiculite substrate. For M+ treatments, 200 g of live AMF inoculum (consisting of AMF spores, extraradical hyphae, colonized maize root fragments, and the propagation substrate) was applied directly around the seedling roots using a layered “sandwich” method to maximize root-inoculum contact. For M- treatments, 200 g of the exact same inoculum, which had been sterilized via autoclaving (121 °C, 0.1 MPa for 2 h) to completely inactivate the AMF, was applied identically to maintain consistent substrate and baseline nutrient conditions. At the time of transplantation, 6 g of a tobacco-specific compound fertilizer (N-P_2_O_5_-K_2_O = 10-10-25) was dissolved in water and uniformly applied to each pot as a base fertilizer. Throughout the experiment, watering, fertilization, weeding, and pest management were consistently maintained across all treatments.

### 2.3 Plant and soil sampling

#### 2.3.1 Plant harvest and measurement of growth indicators

Forty-five days after tobacco transplanting, agronomic traits were evaluated according to the “YC/T 142–2010 Method for Investigating and Measuring Agronomic Traits of Tobacco”. Photosynthetic parameters including net photosynthetic rate (*Pn*), transpiration rate (*Tr*), stomatal conductance (*Gs*), and intercellular CO_2_ concentration (*Ci*), were measured on identical leaf segments using a portable photosynthesis analyzer (LI-6400, LI-COR, USA). Whole plants were removed from the soil, cleaned, and gently blotted dry. Leaf samples (5 g per plant) were flash-frozen in liquid nitrogen for 30 minutes and stored at -80°C for subsequent analysis of antioxidant enzyme activities and biochemical composition. Fresh weights of aboveground and belowground parts were recorded.

#### 2.3.2 Root morphology and colonization assessment

A representative subsample of the fresh roots was carefully washed clean. Root morphological parameters (total length, average diameter, surface area, volume, tip count) were scanned using an Epson Expression 12000XL scanner and analyzed with WinRHIZO Pro software (Regent Instruments Inc., Canada). Another root subsample was cleared with 10% KOH and stained with 0.05% acid fuchsin in lactoglycerol. The percentage AMF colonization was quantified using the gridline intersect method under a compound microscope, examining at least 200 intersects per sample.

#### 2.3.3 Soil sampling

Rhizosphere soil—defined as the soil closely adhering to roots after gentle shaking was collected, sieved through a 2-mm mesh to remove roots and debris, and divided into two portions. One portion was immediately stored at -80 °C for microbial community analysis and allelochemical assays. The other portion was air-dried at room temperature, ground, and passed through a 0.15 mm (100-mesh) sieve for analysis of soil chemical properties and enzyme activities.

#### 2.3.4 Plant antioxidant system and nutrient analysis

After 45 days of tobacco transplantation, fresh samples were collected, and superoxide dismutase (SOD), peroxidase (POD), catalase (CAT), and phenylalanine ammonia-lyase (PAL) activities, as well as contents of malondialdehyde (MDA) and proline (PRO) were determined using commercial assay kits (Greis Bio, China) following the manufacturer’s protocols. Dried plant tissues (shoot and root) were ground to a fine powder. Total nitrogen (N) content was determined by the Kjeldahl method. Total phosphorus (P) and potassium (K) contents were measured after HNO_3_-HClO_4_ digestion, using the molybdenum-blue colorimetric method for P and flame photometry for K.

#### 2.3.5 Soil agrochemical properties and enzyme activities

Air-dried soil samples were used to determine pH by water extraction, soil organic matter was quantified through potassium dichromate titration. Soil alkaline hydrolysable N content was measured with KCl extraction and indophenol blue colorimetry, available P by the molybdenum-antimony colorimetric method, and rapidly available K using flame photometry. Total N was determined with the Kjeldahl method, total P via perchloric acid digestion, and total K through flame photometry. Soil enzyme activities, including sucrase (SC), Catalase (CAT), acid phosphatase (ACP), and polyphenol oxidase (PPO), were measured using Grace Bio-Labs kits.

#### 2.3.6 Soil microbial diversity and allelochemicals

Total genomic DNA was extracted from frozen rhizosphere soil samples using the Power Soil DNA Isolation Kit. The V3-V4 region of the bacterial 16S rRNA gene and the ITS2 region of the fungal ITS rRNA gene were amplified and sequenced on an Illumina NextSeq platform (Majorbio Bio-Pharm Technology, Shanghai, China). For the high-throughput sequencing of microbial communities, 6 independent biological replicates were randomly selected from the 10 replicates of each treatment group. Bioinformatic analysis was performed on the Majorbio Cloud Platform (https://www.majorbio.com). For allelochemical analysis, frozen soil samples were freeze-dried. Target compounds (benzoic acid, ferulic acid, cinnamic acid, p-hydroxybenzoic acid, vanillic acid, p-coumaric acid, myristic acid) were extracted and quantified using liquid chromatography-mass spectrometry (LC-MS) by Wuhan Punes Testing Technology Co. Ltd., China. LC-MS analysis was performed using an Agilent 1260 high-performance liquid chromatograph coupled with a 6420A mass spectrometer. Chromatographic separation was achieved on an Agilent Poroshell 120 EC-C18 column (2.7 µm, 3 × 100 mm), with the column temperature maintained at 35 °C. The mobile phase consisted of an aqueous solution containing 0.1% formic acid (A) and acetonitrile (B), utilizing a gradient elution mode. The mass spectrometer operated in positive electrospray ionization (ESI) mode, employing multiple reaction monitoring (MRM) technology to analyze specific ions of the allelochemicals. The specific elution gradient is shown in the table below.

**Table:** Table. Elution gradient of allelochemicals used in the experiment.

Time(min)	A%	B%
0.01	90	10
2	80	20
6	10	90
10	1	99
9.10	90	10
15	Stop	

### 2.4 Data analysis

All data are presented as mean ± standard deviation (SD). Two-way analysis of variance (Two-way ANOVA) was performed using SPSS Statistics 23.0 (IBM Corp., USA) to evaluate the main effects of soil type and AMF inoculation, as well as their interaction, on all measured variables. Prior to conducting the analysis of variance, all data were tested for normality using the Shapiro-Wilk test and for homogeneity of variances using Levene’s test. Data that did not meet these assumptions were log-transformed prior to analysis to satisfy the requirements for parametric testing. For comparisons between the two soil types under non-inoculated conditions (to characterize the baseline effect of continuous cropping), independent samples t-tests were used. Graphs were generated using Origin 2022 (OriginLab Corp., USA). Differences were considered statistically significant at *p* < 0.05.

## 3 Results

### 3.1 AMF colonization rate in tobacco roots under different treatments

**Figure 1:**
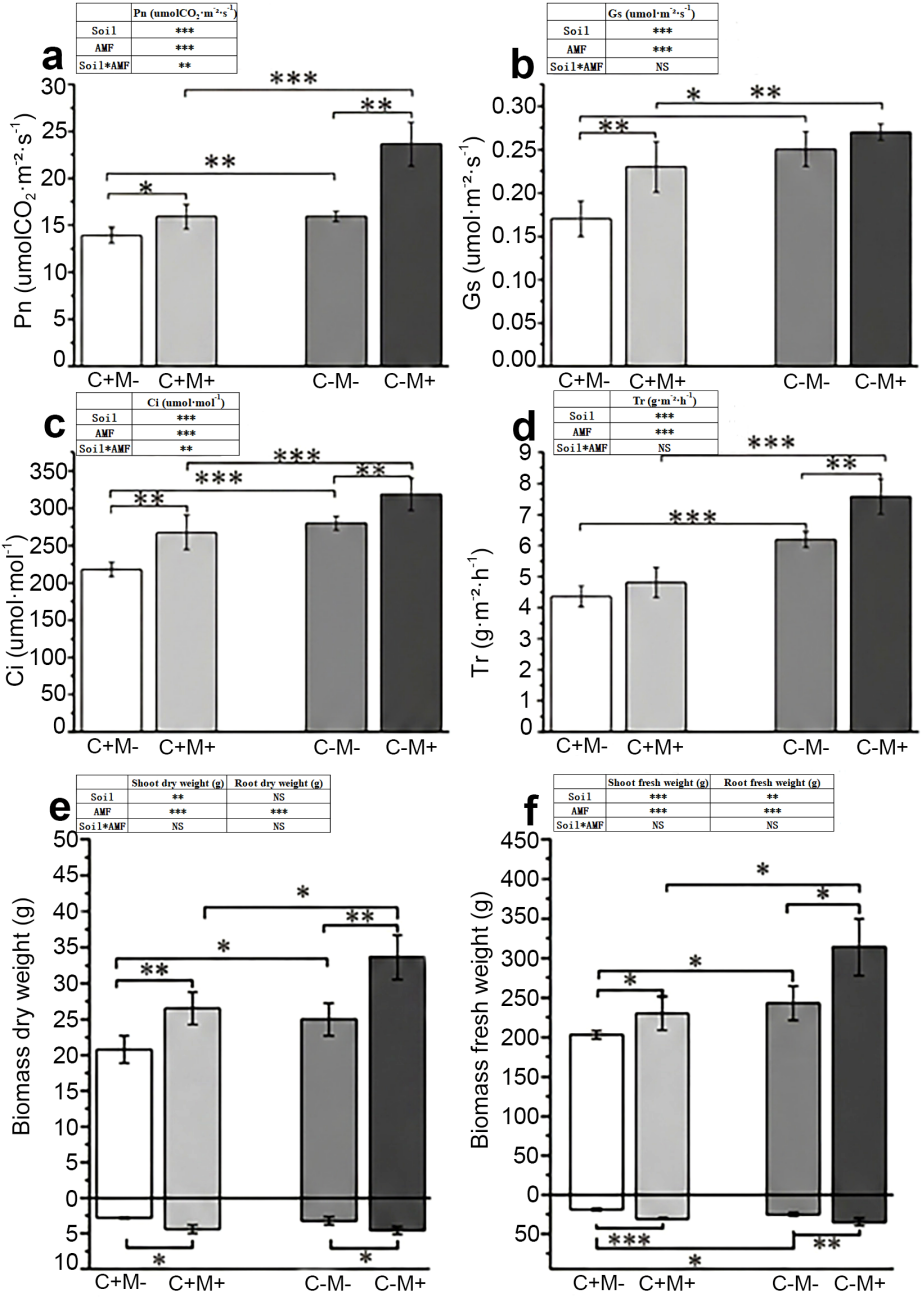
Effects of different inoculation treatments and soil types on photosynthetic parameters and biomass **(a)** Net photosynthetic rate (Pn); **(b)** Stomatal conductance (Gs); **(c)** Intercellular CO_2_ concentration (Ci); **(d)** Transpiration rate (Tr); **(e)** Shoot dry weight; **(f)** Shoot fresh weight. * Indicates a significant difference between the two groups (*p < 0.05, ** *p* < 0.01, *** *p* < 0.001). C+, Soil continuously cropped with tobacco for five years; C-, Non-continuous cropping soil; M+, Inoculated with *Funneliformis mosseae*; M-, Non-inoculated control.

Successful colonization by *F. mosseae* was established in tobacco roots 45 days after transplantation across all treatments, forming arbuscules, hyphae, vesicles, and hyphal coils (**Figure 1**). In continuous and non-continuous cropping soils, total colonization rates of AMF-inoculated roots reached 68.8% and 74.4%, respectively, compared to significantly lower rates of 27.8% and 28.4% in non-inoculated controls. Moreover, two-factor analysis revealed a significant interactive effect of AMF inoculation and soil type on the colonization rates of specific structures of arbuscules, hyphae, and hyphal coils (**Table 2**). The specific nature of this interaction indicates a stress-induced structural adaptation of the symbiosis. In the continuous cropping soil (C+), which is characterized by allelochemical stress and poor nutrient bioavailability, AMF colonization preferentially formed arbuscules—the primary physiological sites for host-fungus nutrient exchange—to actively meet the host’s urgent need for stress alleviation. Conversely, in the relatively healthier non-continuous cropping soil (C-), the symbiosis significantly favored the proliferation of hyphal coils, which function primarily as fungal storage and vegetative spread organs.

**Table 2:** After 45 days of transplanting, the colonization rate of AMF in flue-cured tobacco of each treatment group (mean ± standard error, n = 3).

Treatments		Mycorrhizal colonization intensity Two- way				
		Arbuscule (%)	Hyphae (%)	Hyphal coil (%)	Vesicle (%)	Total (%)
C+	M-	13.20 ± 0.8	8.2 ± 0.84	6.4 ± 0.89	4.4 ± 1.14	27.8 ± 3.11
	M+	62.60 ± 1.34***	17.8 ± 0.84***	11.4 ± 1.52***	4.6 ± 1.67	68.8 ± 3.27***
C-	M-	***14.20 ± 0.45	9.4 ± 0.9	4.6 ± 0.55	**5.6 ± 1.14	*28.4 ± 2.07
	M+	***71.20 ± 1.79***	***17 ± 0.71	**14.4 ± 0.55***	**8.2 ± 1.48	**74.4 ± 1.34***
Two-factor analysis	Soil	***	NS	**	***	***
	AMF	***	**	***	NS	***
	Soil*AMF	***	***	**	NS	NS

*Indicates a significant difference between treatment groups under independent samples T-test (*p < 0.05, **p < 0.01, ***p< 0.001). For two-way ANOVA, * indicates a significant effect of the independent variable on the dependent variable under LSD, two-tailed test (NS: *p* > 0.05, * *p* < 0.05, **p < 0.01, ***p < 0.001). The asterisk on the right indicates the difference between treatment groups with and without AMF inoculation under the same soil conditions, on the left indicates the difference between continuous cropping soil and non-continuous cropping soil under the same inoculation conditions. Detailed Two-way ANOVA results, including F-values and effect sizes (η_p_^2^), are provided in **Supplementary Table 8**.

### 3.2 Effects of continuous cropping soil and AMF-inoculated on tobacco growth

Continuous cropping soil impaired root growth, whereas AMF inoculation effectively mitigated these negative effects. In non-continuous cropping soils, AMF-inoculated plants (C-M+) exhibited significantly greater total root length, surface area, average root diameter, number of root tips and bifurcations relative to the other treatments (**Supplementary Table 1**), indicating enhanced root system development. AMF inoculation significantly increased net photosynthetic rate (*Pn*) and intercellular CO_2_ concentration (*Ci*) compared to non-inoculated plants (**Figure 1**). Non-continuous cropping soil alone also elevated photosynthetic parameters (P_n_, Gs, Ci, Tr), and two-way ANOVA revealed significant soil and AMF interactions for Pn and Ci, suggesting synergistic effects. Across soil types, AMF inoculation significantly increased plant height, stem circumference, leaf dimensions, leaf number (**Supplementary Figure 2**; **Supplementary Table 2**), and biomass (**Figure 1**). Additionally, non-continuous cropping soil similarly enhanced these parameters compared to continuous cropping soil, although underground dry weight did not differ significantly (*p* > 0.05).

### 3.3 Effects of continuous cropping soil and AMF on N, P and K content in tobacco

**Figure 2:**
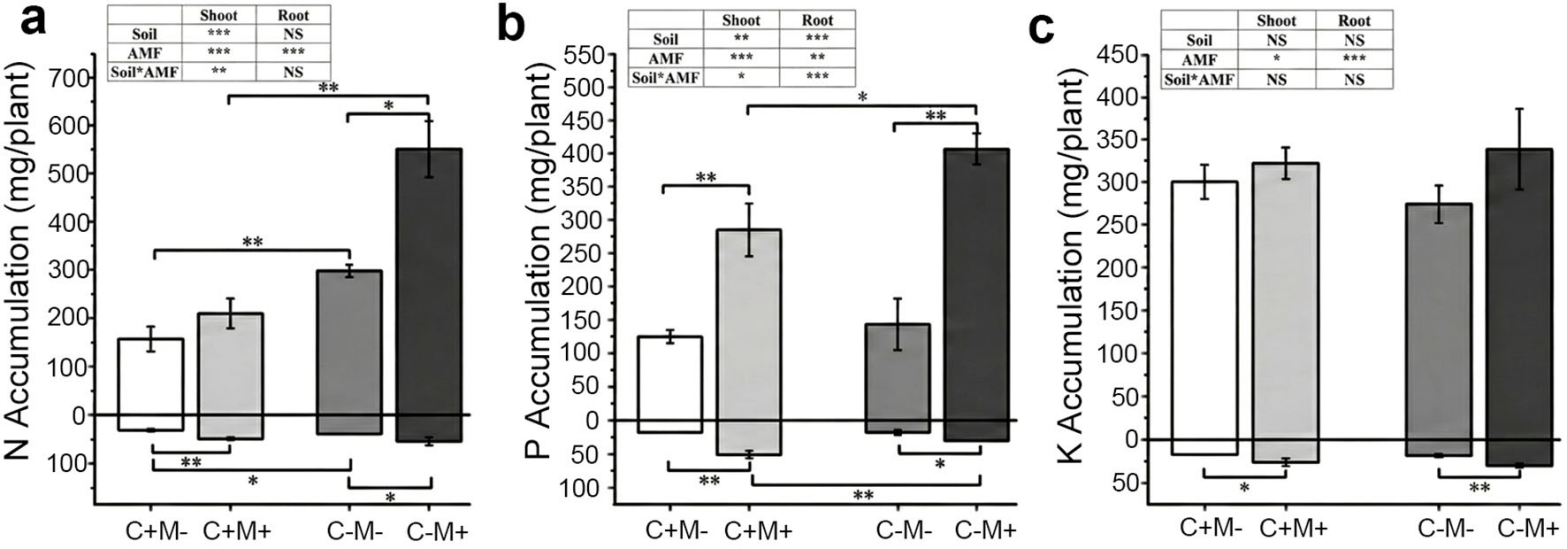
Effects of AMF inoculation on nutrient accumulation in tobacco tissues. The bar charts illustrate the accumulation of **(a)** Nitrogen (N), **(b)** Phosphorus (P), and **(c)** Potassium (K) in the shoot and root tissues of tobacco plants. Bar heights represent the mean values, and error bars indicate the standard deviation. Asterisks denote statistically significant differences between specific treatment pairs (*p < 0.05, **p < 0.01, ***p < 0.001).

Compared to continuous cropping soil, non-continuous cropping soil significantly increased N content in both the above-ground and underground tobacco tissues. Across soil types, AMF inoculation significantly increased accumulation of N, P, and K in roots and enhanced N and P contents in above-ground tissues. However, AMF inoculation had no significant effect on K accumulation in the above-ground tissues (**Figure 2**). These findings demonstrate that AMF inoculation improves nutrient acquisition, particularly N and P.

### 3.4 Effects of continuous cropping soil and AMF on antioxidant enzyme activities and substance contents in tobacco leaves and roots

**Figure 3:**
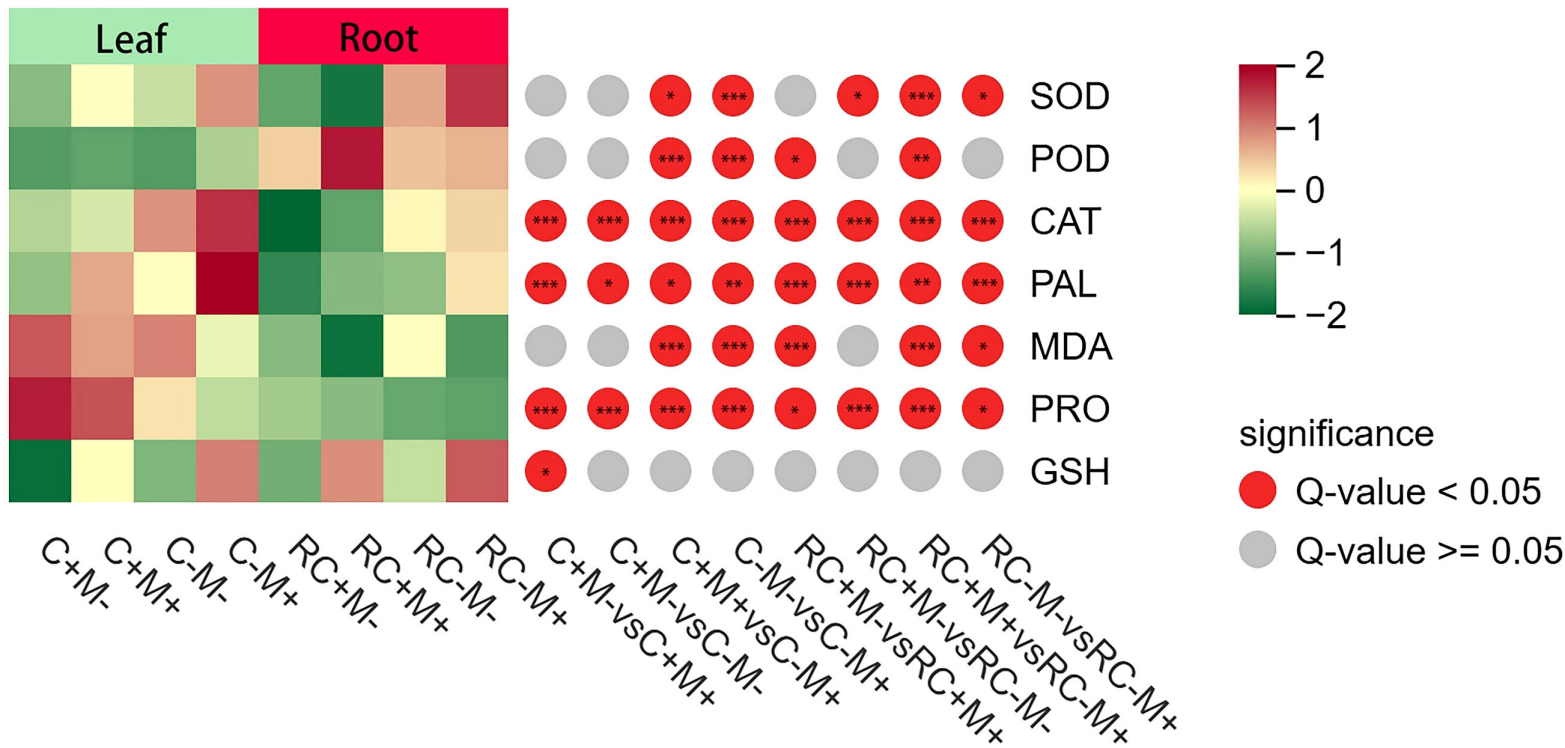
Effects of AMF and continuous cropping soil on antioxidant defense system and stress resistance indexes of flue-cured tobacco leaves and roots. The red circle on the right indicates a significant difference between the two groups (p<0.05), and the gray indicates no significant difference. * Indicates a significant difference between the two groups (*p < 0.05, **p < 0.01, ***p < 0.001). SOD (Superoxide Dismutase, u/g); POD (Peroxidase, ΔOD470/min/g); CAT (Catalase, umol/min/g); PAL (Phenylalanine Ammonia-lyase, ΔOD290/h/g); MDA (Malondialdehyde, nmol/g); PRO (Proline, ug/g); GSH (Glutathione, umol/g).

Continuous cropping induced oxidative stress, as reflected by elevated MDA and PRO levels and reduced activities of key antioxidant enzymes. AMF inoculation significantly enhanced the plant’s antioxidant defense system (**Figure 3**; **Supplementary Tables 3**, **S4**). In leaves, AMF significantly increased SOD, POD, CAT, and PAL activities compared to non-inoculated plants. Furthermore, non-continuous cropping soils increased SOD, CAT, and PAL activities (*p* > 0.05). Two-way ANOVA confirmed that AMF under continuous cropping significantly increased POD, MDA, PRO, and CAT activities (**Supplementary Table 3**).

In roots, AMF inoculation significantly increased POD, CAT, and PAL activities. Non-continuous cropping soils markedly increased SOD, CAT, and PAL activities, as well as POD activity and glutathione (GSH) content (*p* > 0.05). Significant soil and AMF interactions were observed for SOD, POD, CAT, and PAL activities, (**Supplementary Table 4**), indicating the protective effects of AMF are amplified in stressed soils.

### 3.5 AMF improves soil chemical properties and enzyme activities

**Figure 4:**
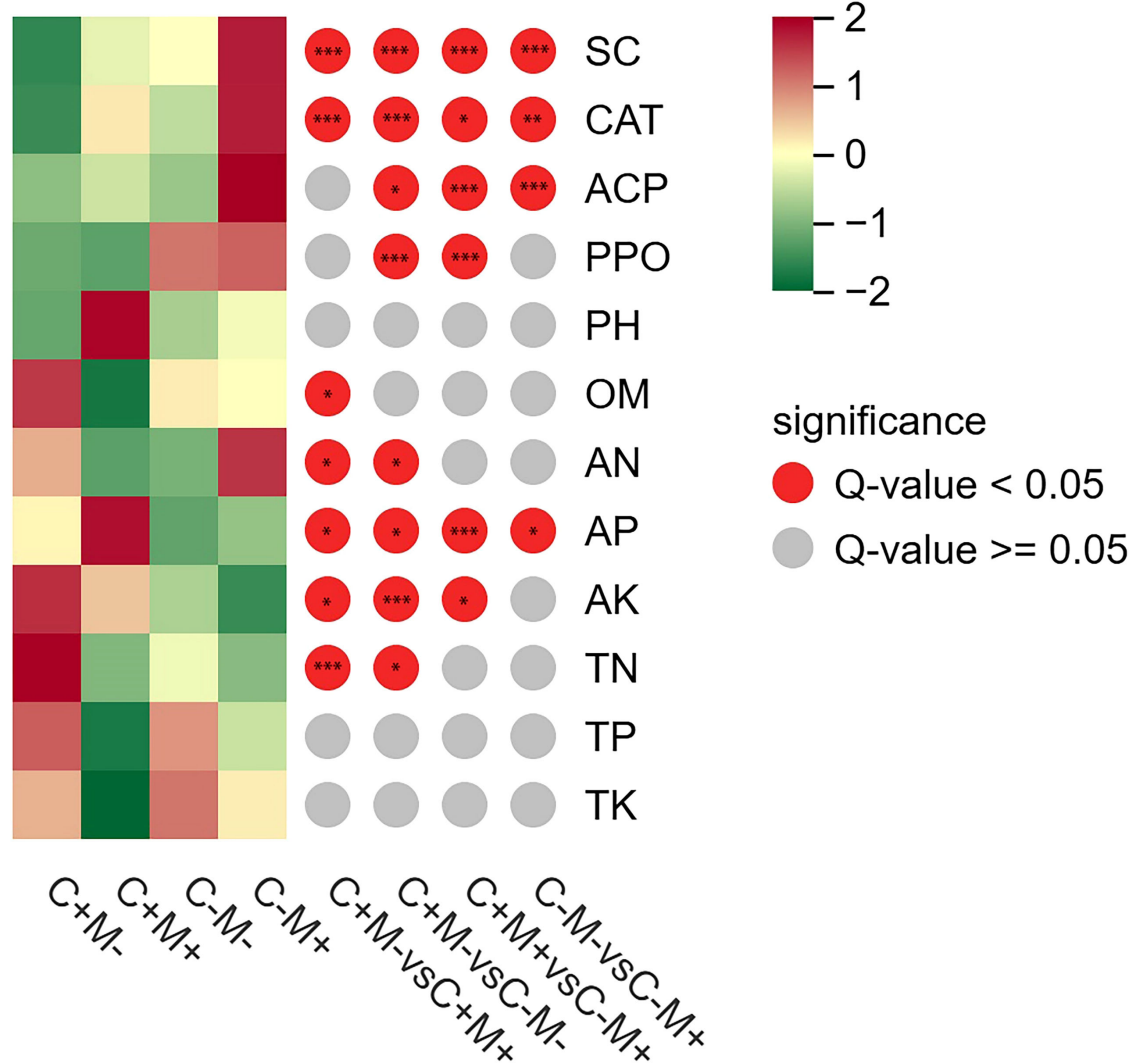
Effects of AMF and continuous cropping soil on the physicochemical properties and soil enzyme activity of rhizosphere soil of tobacco. Heatmap illustrating the effects of AMF inoculation and continuous cropping on the physicochemical properties and enzyme activities of tobacco rhizosphere soil. The color gradient (from green to red) represents the normalized values (Z-scores) of soil parameters, ranging from lowest to highest. The circles on the right denote the results of significance testing between treatment groups, where red circles indicate a significant difference (*p*< 0.05) and gray circles indicate no significant difference. * Indicates a significant difference between treatment groups under independent samples Ttest (* *p* < 0.05, ** *p* < 0.01, *** *p* < 0.001). OM, Organic Matter; AN; Alkaline Hydrolyzable Nitrogen; AP, Available Phosphorus; AK, Available Potassium; TN, Total Nitrogen; TP, Total Phosphorus; TK, Total Potassium; SC, Sucrase, mg/d/g; CAT, Catalase, umol/h/g; ACP, Acid Phosphatase, umol/h/g; PPO, Polyphenol Oxidase, nmol/h/g.

Analysis of soil agrochemical properties revealed that pH, total P, and total K contents did not differ significantly among treatments (**Figure 4**). While AMF inoculation decreased soil organic matter, available K, and total N, P, and K contents (*p* > 0.05). Two-way ANOVA demonstrated that significant soil and AMF interactions for organic matter, available P, and total N contents (**Supplementary Table 5**).

AMF inoculation significantly increased soil CAT and SC activities in both soil types, and under non-continuous cropping, significantly increased ACP activity. Conversely, continuous cropping soil exhibited substantially reduced CAT, ACP, SC, and POD activities compared with non-continuous cropping soils (**Figure 4**). Two-way ANOVA analysis showed that continuous cropping and AMF interactions significantly influenced soil organic matter, available P, total N contents, and the activities of ACP and SC (**Supplementary Table 6**).

Apparent nutrient utilization efficiency (REU) for N, P, and K was significantly lower in continuous cropping soil but was markedly enhanced by AMF inoculation in both soil types (**Table 3**). Significant soil and AMF interactions were observed for N and P utilization efficiency, indicating the positive effects of AMF are particularly pronounced under continuous cropping stress.

**Table 3:** Effects of different inoculation treatments and soil types on the efficiency of fertilizer uptake by tobacco plants.

Treatments		Fertilizer absorption efficiency (%)		
		N	P	K
C+	M+	23.96 ± 3.90	29.21 ± 2.86***	6.327 ± 0.33
	M-	17.86 ± 2.70	12.76 ± 0.81	5.51 ± 0.80
C-	M+	***69.22 ± 7.03*	***49.26 ± 2.67***	**10.77 ± 1.46
	M-	***38.94 ± 1.54	18.07 ± 4.11	**8.54 ± 0.60
Soil	P	***	***	*
AMF	P	***	***	***
Soil*AMF	P	**	**	NS

The apparent nutrient utilization rate (REU, %) is calculated as follows: REU (%) = (U – U0) F 100 %, where U represents the amount of nitrogen (mg/plant), phosphorus (mg/plant), and potassium (mg/plant) nutrients absorbed by the crop under fertilization treatment, U0 represents the amount of nitrogen (mg/plant), phosphorus (mg/plant), and potassium(mg/plant) nutrients absorbed without fertilization, and F represents the amount of nitrogen (mg/plant), phosphorus (mg/plant), and potassium (mg/plant) nutrients applied (Bu et al., 2025). * Indicates a significant difference between treatment groups under independent samples T-test (*p < 0.05, **p < 0.01, ***p < 0.001). In the two-factor analysis, * indicates a significant effect of the independent variable on the dependent variable under LSD, two-tailed test (NS: *p* > 0.05, *p < 0.05, **p < 0.01, ***p < 0.001). The asterisk on the right indicates the difference between treatment groups with and without AMF inoculation under the same soil conditions, and the asterisk on the left indicates the difference between continuous cropping soil and noncontinuous cropping soil under the same inoculation conditions. Detailed Two-way ANOVA results, including F-values and effect sizes (hp 2), are provided in **Supplementary Table 9**.

### 3.6 Effects of soil type and AMF on microbial diversity in the tobacco rhizosphere soil

#### 3.6.1 Analysis of bacterial and fungal community diversity

**Figure 5:**
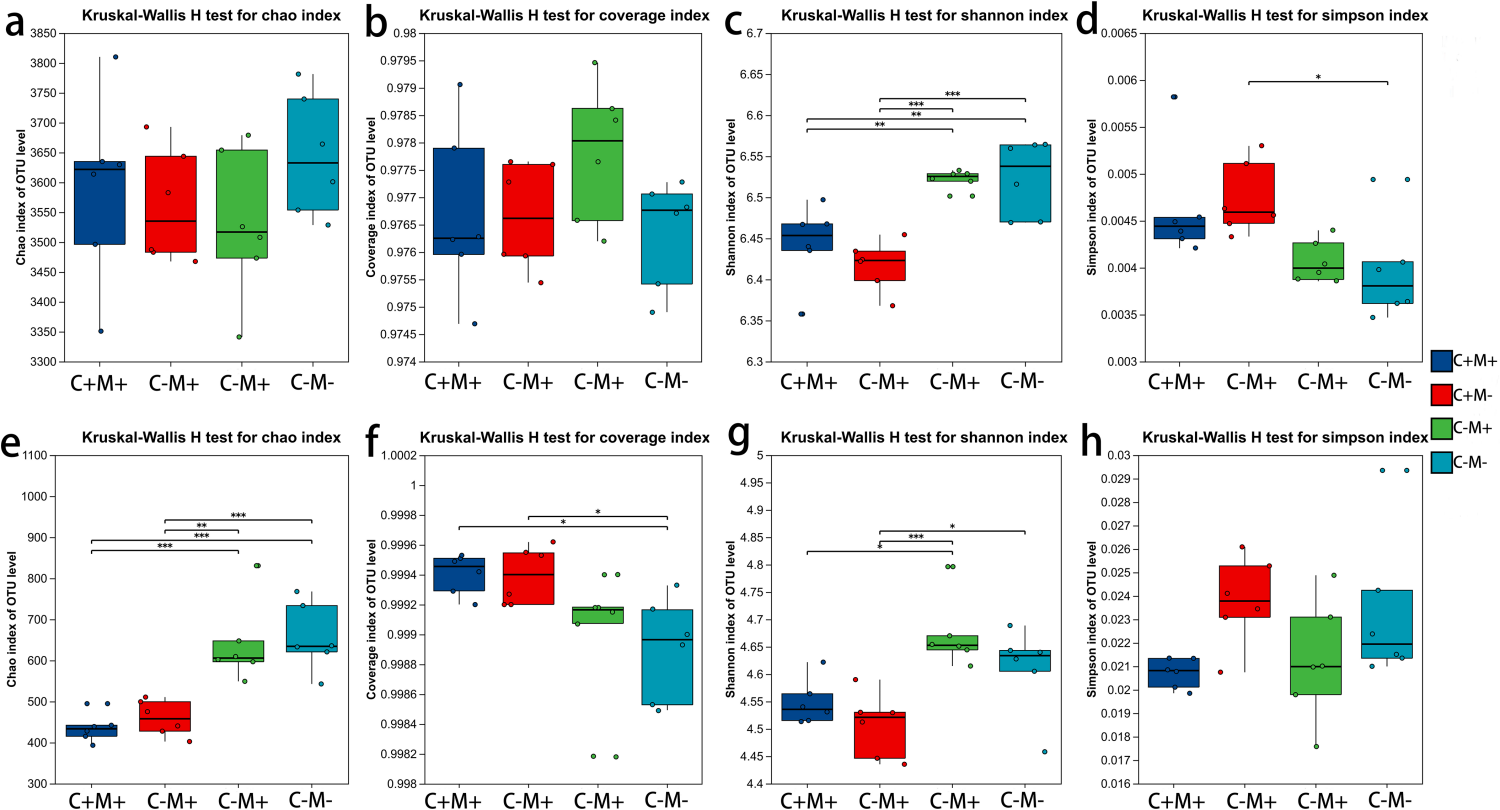
Differences in α-diversity indices among different treatment groups. **(a–d)** Box plots showing the Chao, Coverage, Shannon, and Simpson indices for bacterial communities, respectively. **(e–h)** Box plots showing the Chao, Coverage, Shannon, and Simpson indices for fungal communities, respectively. Significant differences are indicated by * (*p* < 0.05), ** (*p* < 0.01), and *** (*p* < 0.001).

**Figure 6:**
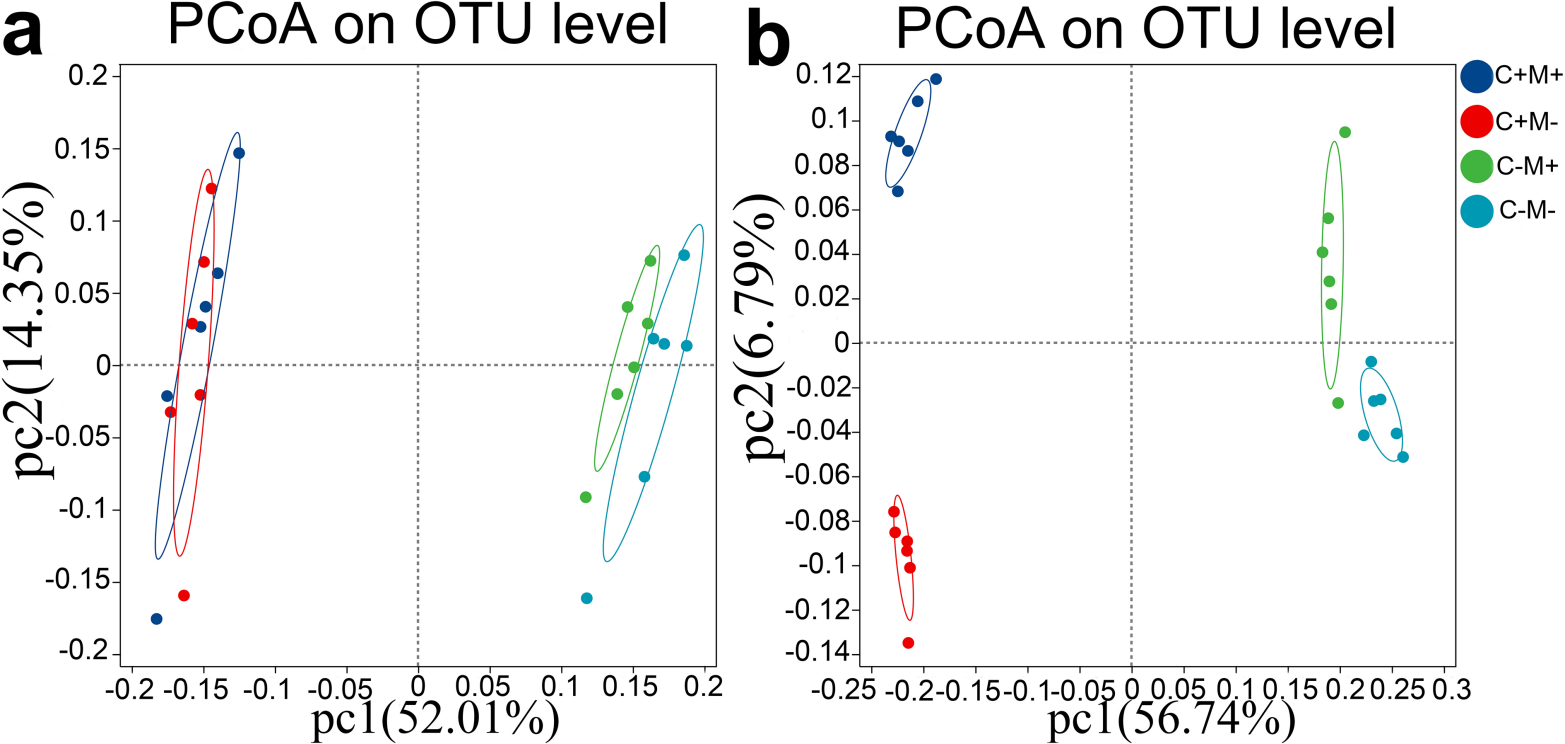
PCoA (principal coordinates analysis) of bacterial **(a)** and fungal **(b)** communities in the rhizosphere of tobacco plants under different inoculation treatments, based on OTU level.

In continuous cropping soil, the Chao, Coverage, and Shannon indices of bacterial communities increased following AMF inoculation (p > 0.05). In non-continuous cropping soil, AMF inoculation increased Coverage and Shannon indices (**Figures 5a–d**); under continuous cropping soil conditions, inoculation with AMF resulted in a decrease in the Simpson index; under non-continuous cropping soil conditions, it led to a decrease in the Chao and Simpson indices, while increasing the Coverage and Shannon indices (**Figures 5e–h**). Principal coordinate analysis (PCoA) based on Bray-Curtis distance revealed clear separation of both bacterial and fungal communities among treatment groups (**Figures 6a,b**). AMF exerted a stronger effect on fungal community structure than on bacterial communities, as indicated by greater dispersion among fungal samples.

#### 3.6.2 Shifts in microbial community composition

**Figure 7:**
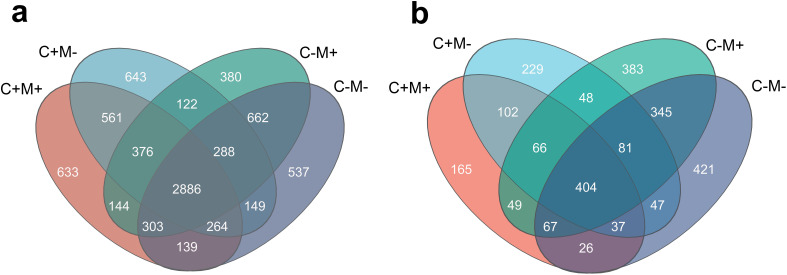
Venn diagrams of bacteria **(a)** and fungi **(b)** in tobacco rhizosphere soil under different inoculation treatments.

Total bacterial OTUs across all treatments were 2886, while fungal OTUs totaled 404 (**Figure 7**). Continuous cropping increased bacterial OTUs but reduced fungal OTUs. AMF inoculation decreased OTU numbers for both bacterial and fungal communities, suggesting selection for specific functional taxa rather than overall richness.

At the phylum level, dominant bacterial phyla across all treatments were Proteobacteria, Actinobacteriota, Acidobacteriota, Chloroflexi, and Gemmatimonadota. In continuous cropping soil, AMF inoculation reduced Actinobacteriota and Gemmatimonadota and increased Proteobacteria, Acidobacteriota, and Chloroflexi. In non-continuous cropping soil, AMF inoculation decreased *Proteobacteria* and Actinobacteriota but increased Acidobacteriota, Chloroflexi, and Gemmatimonadota. Continuous cropping alone decreased Actinobacteriota and Acidobacteriota. Regarding fungal phyla, the dominant groups in the soil were Ascomycota, Mortierellomycota, Chytridiomycota, Basidiomycota, and Olpidiomycota, ranked by relative abundance. AMF inoculation reduced Mortierellomycota and Basidiomycota and increased Ascomycota and Chytridiomycota (**Supplementary Figure 3**). Among the top 15 bacterial genera in tobacco rhizosphere soil, those with average relative abundance greater than 2 percent included Vicinamibacterales, Vicinamibacteraceae, Gemmatimonadaceae, Gaiellales, Sphingomonas, Bacillus, and Roseiflexaceae. Under continuous and non-continuous cropping, AMF inoculation increased Vicinamibacterales and Sphingomonas and decreased Gemmatimonadaceae and Bacillus. In continuous cropping soil, the relative abundance of Vicinamibacterales and Vicinamibacteraceae were decreased, while Gemmatimonadaceae, Gaiellales, Sphingomonas, and Bacillus were increased.

**Figure 8:**
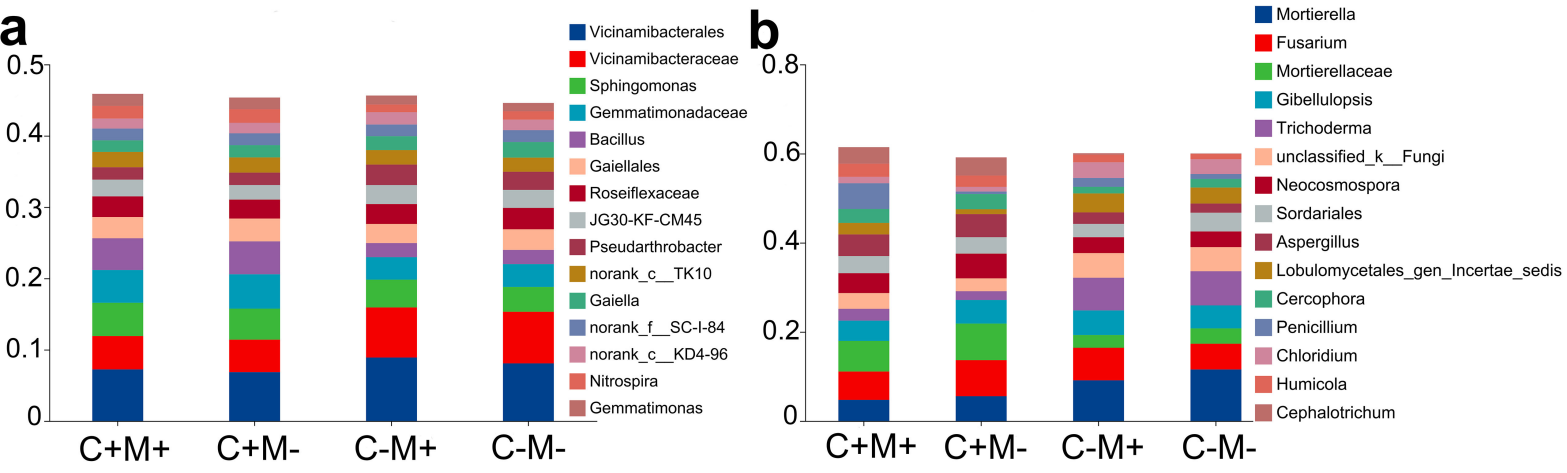
Community composition and abundance of bacteria **(a)** and fungi **(b)** at the genus level in tobacco rhizosphere soil.

At the genus level, within the top 15 fungal genera, dominant taxa included *Fusarium*, *Mortierella*, *Gibellulopsis*, *Neocosmospora*, *Trichoderma*, *Aspergillus*, and *Cercophora*. In continuous cropping soil, AMF inoculation reduced *Fusarium*, *Mortierella*, *Gibellulopsis*, and *Neocosmospora* and increased *Trichoderma*. Conversely, in non-continuous cropping soil, AMF inoculation decreased *Mortierella* and *Trichoderma* and increased *Fusarium*, *Gibellulopsis*, and *Neocosmospora*. Under the same inoculation conditions, continuous cropping soil showed a greater reduction in *Mortierella* and *Trichoderma* and increased *Neocosmospora*, *Aspergillus*, and *Cercophora* (**Figure 8**).

To rigorously identify the specific microbial shifts driven by AMF, LEfSe analysis was performed to detect statistically significant biomarkers (**Supplementary Figure 4**). In the bacterial community, AMF inoculation under continuous cropping (C+M+) significantly enriched beneficial taxa at the genus level, specifically Sphingomonas (LDA score = 3.76, p< 0.05) and Gemmatimonas (LDA score = 3.41, p< 0.05). Conversely, non-inoculated continuous cropping soil (C+M-) was characterized by a significant enrichment of the phylum Firmicutes (LDA score = 4.31, p< 0.05) and the genus Bacillus (LDA score = 4.29, p< 0.05; **Supplementary Figure 5**). For the fungal community, the non-inoculated continuous cropping soil (C+M-) exhibited a significant accumulation of key phytopathogens, including Fusarium (LDA score = 4.21, p< 0.05) and Aspergillus (LDA score = 4.31, p< 0.05). AMF inoculation (C+M+) successfully restricted these pathogens while significantly enriching the phylum Ascomycota (LDA score = 4.86, p< 0.05) and the genus Penicillium (LDA score = 4.52, p< 0.05; **Supplementary Figure 5**).

**Figure 9:**
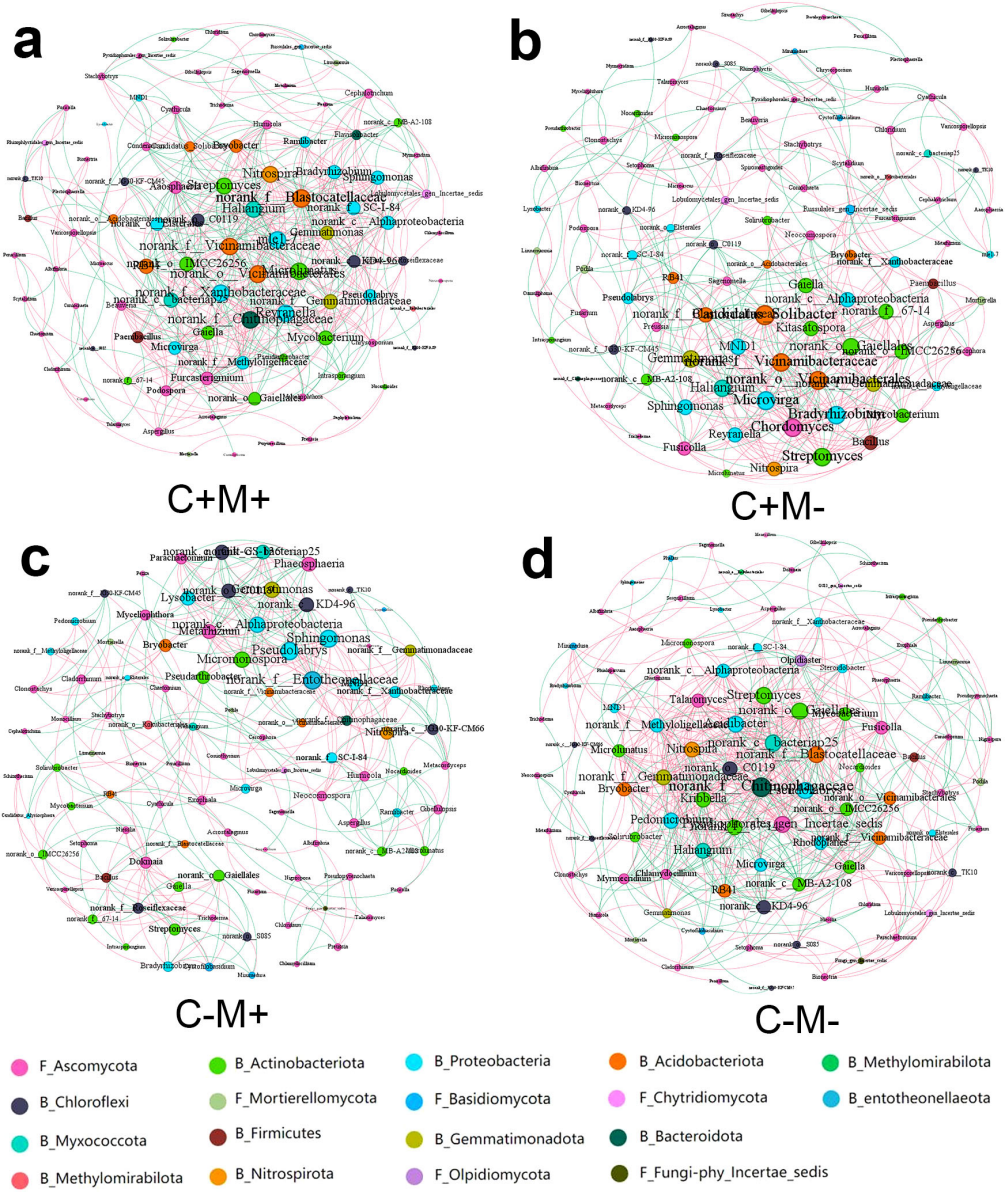
Co-occurrence network diagrams of bacteria and fungi in soil under different treatment groups **(a–d)**. In Figures a, b, c, and d, connections indicate statistically significant (*p* < 0.01) strong positive correlations (red, Spearman’s *ρ* > 0.6) or negative correlations (blue, Spearman’s *ρ*< -0.6). The size of each node is proportional to the number of connecting edges, and nodes of the same color belong to the same phylum. The thickness of each connection between two nodes is proportional to the value of the Spearman correlation coefficient *ρ* > 0.6 or *ρ*< -0.6.

A co-occurrence network analysis of the top 50 genera indicated that the principal nodes comprised 12 bacterial and 6 fungal phyla, with Ascomycota, Actinobacteriota, and Proteobacteria being the most predominant (**Figures 9a–d**). Under non-inoculated conditions, network complexity—measured by average clustering coefficient, graph density, average degree, and edge count—was lower in continuous cropping soil than in non-continuous cropping soil. However, AMF inoculation reversed this trend: it increased network complexity in continuous cropping soil while reducing it in non-continuous cropping soil (**Supplementary Table 7**). These findings suggest that AMF specifically enhances microbial interaction complexity under continuous cropping stress. Notably, positive correlations dominated across all networks, suggesting a high degree of co-occurrence among soil bacteria and fungi. While this may imply potential synergistic interactions, it could also reflect shared ecological niches or similar adaptation strategies to the dynamic rhizosphere environment.

#### 3.6.3 Functional prediction of microbial communities

**Figure 10:**
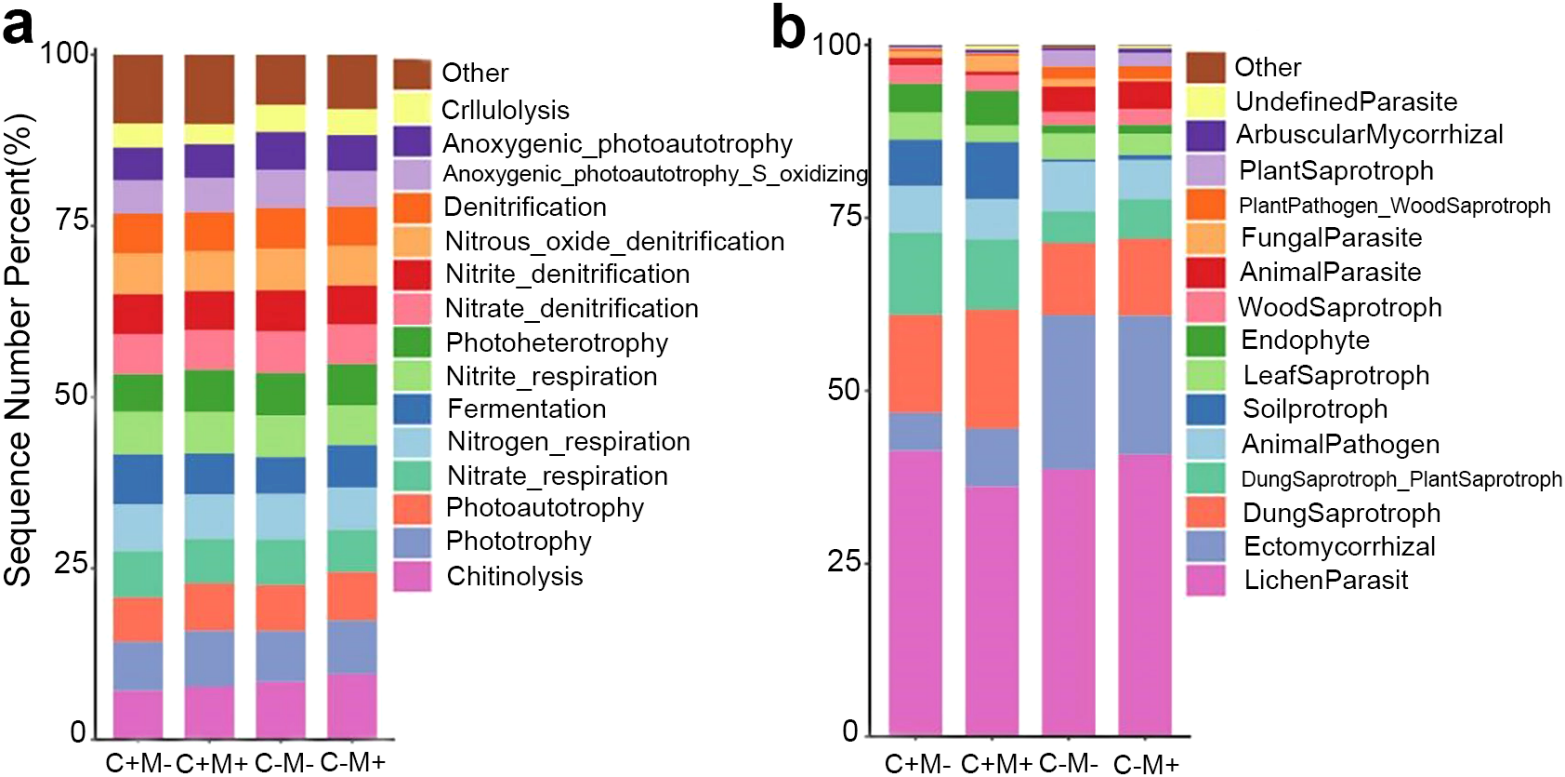
Comparison of bar graphs of the relative abundances of the functions of bacteria **(a)** and fungi **(b)** under different treatments.

Bacterial ecological functions were predicted using the FAPROTAX database, assigning sequences to 29 functional categories. The predominant functions included degradation of aromatic compounds, photoheterotrophy, photoautotrophy, N respiration, and nitrate respiration (**Figure 10a**). Across both soil types, these five categories were more abundant in non-continuous cropping soil. AMF inoculation was associated with an enrichment of bacterial taxa predicted to be involved in chitin degradation, photoautotrophy, and photoheterotrophy specifically in continuous cropping soil, while the relative abundance of taxa associated with nitrate respiration was reduced.

Fungal functional groups were predicted using FUNGuild and categorized into three major trophic modes: saprotrophic, pathogenic, and symbiotic fungi (**Figure 10b**). Based on FUNGuild predictions, AMF inoculation consistently correlated with an increased relative abundance of taxa assigned to symbiotic fungal guilds and a decrease in putative animal pathogens. In continuous cropping soil specifically, AMF increased soil saprotrophs and endophytes, while decreasing lichen parasites—a trend that reversed the pattern observed in non-inoculated controls. Dung saprotrophs increased with AMF inoculation in both soil types, whereas AMF had differential effects on dung saprotroph-plant saprotrophs depending on soil type. Overall, AMF inoculation is suggested to shift the fungal community toward a more symbiotic and saprotrophic, and less pathogenic, functional profile.

### 3.7 Effects of AMF and continuous cropping on allelochemicals in tobacco rhizosphere soil

Continuous cropping significantly increased the concentrations of most measured allelochemicals, including p-hydroxybenzoic acid, benzoic acid, and cinnamic acid (**Table 4**), Conversely, AMF inoculation significantly reduced the levels of hydroxybenzoic acid, coumaric acid, ferulic acid, and myristic acid. Meanwhile, vanillic acid exhibited a non-significant decrease (*p* > 0.05). Notably, benzoic acid and cinnamic acid increased following AMF inoculation, suggesting differential regulation of specific phenolic compounds.

**Table 4:** Effects of different inoculation treatments on the accumulation of phenolic acids in tobacco rhizosphere soil.

Treatments	P-hydroxybenzoic acid(ng/g)	Vanillic acid (ng/g)	P-coumaric acid (ng/g)	Ferulic acid (ng/g)	Benzoic acid (ng/g)	Cinnamic acid (ng/g)	Myristic acid (ng/g)
C+M-	**523.49 ± 7.14***	921.53 ± 24.59	3161.1 ± 59.6***	479.78 ± 20.11**	***14.61 ± 0.71	***116.79 ± 5.76	1411.45 ± 51.59***
C+M+	415.88 ± 18.94	*872.09 ± 32.61	2106.82 ± 87.76	412.25 ± 11.71	21.98 ± 0.65***	***210.25 ± 4.34***	158.22 ± 2.13
C-M-	482.9 ± 11.61***	918.09 ± 39.19**	***7394.54 ± 77.01***	***701.15 ± 24.63***	8.96 ± 0.44	30.49 ± 1.01	**1651.74 ± 69.89*
C-M+	405.27 ± 4.84	809.10 ± 37.44	***5384.48 ± 54.34	***587.55 ± 14.99	**26.03 ± 1.23***	40.81 ± 1.34***	***1511.73 ± 71.49
Soil	**	NS	***	***	NS	***	***
AMF	***	**	***	***	***	***	***
Soil*AMF	*	NS	***	*	***	***	***

* Indicates a significant difference between treatment groups under independent samples T-test (*p < 0.05, **p < 0.01, ***p < 0.001). In the two-factor analysis, * indicates a significant effect of the independent variable on the dependent variable under LSD, two-tailed test (NS: *p* > 0.05, * *p<no><</no>* 0.05, ** *p* < 0.01, *** *p* < 0.001). The asterisk on the right indicates the difference between treatment groups with and without AMF inoculation under the same soil conditions, and the asterisk on the left indicates the difference between continuous cropping soil and non-continuous cropping soil under the same inoculation conditions. Detailed Two-way ANOVA results, including F-values and effect sizes (η_p_^2^), are provided in **Supplementary Table 10**.

### 3.8 Integrative correlation and path analyses.

#### 3.8.1 Correlation between soil properties and allelochemicals

**Figure 11:**
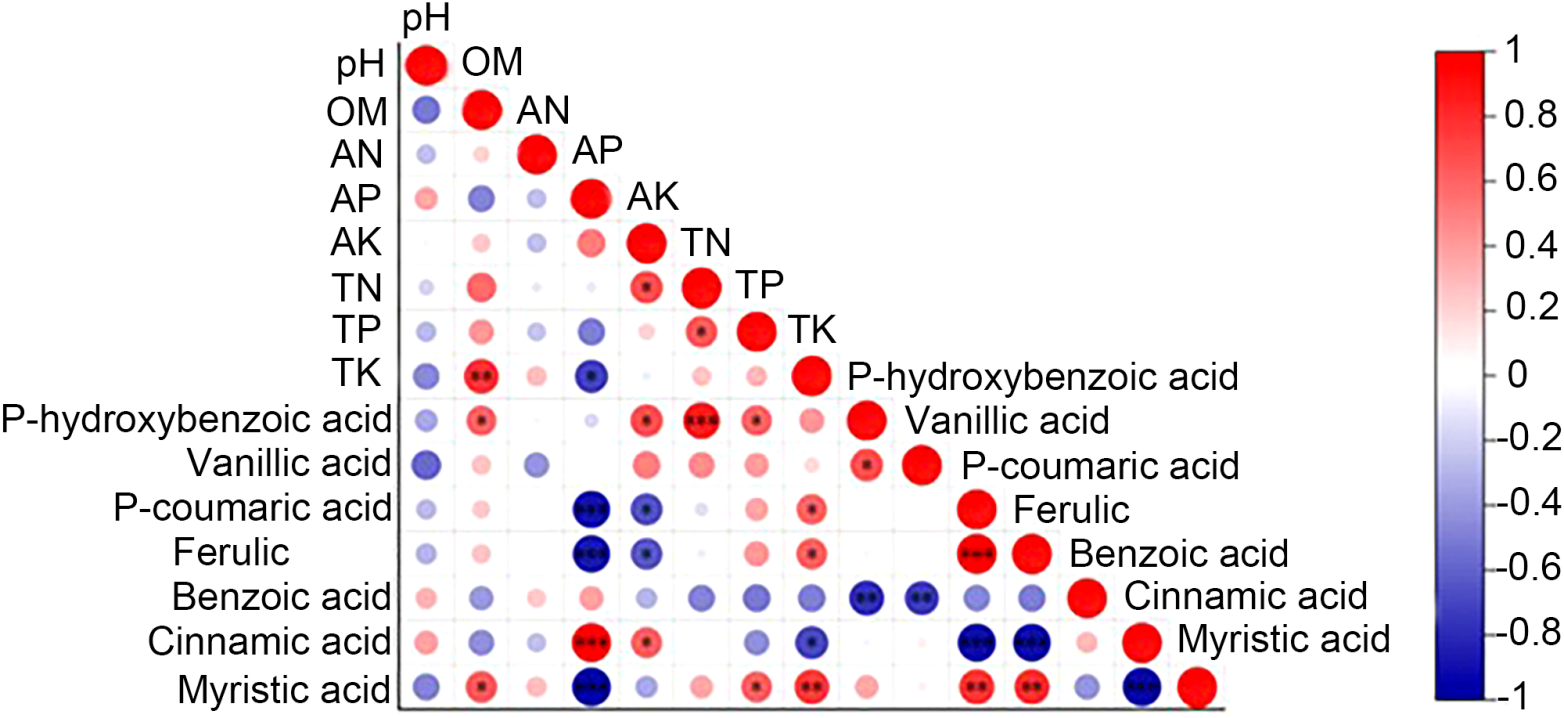
Correlation analysis of agrochemical properties of rhizosphere soil and contents of root exudates of flue-cured tobacco under different inoculation treatments. Red represents positive correlation; blue represents negative correlation. * Indicates a significant correlation between substances (*p < 0.05, **p < 0.01, ***p < 0.001).

Soil organic matter was significantly positively correlated with myristic acid (**Figure 11**). Conversely, available P content showed significant negative correlations with p-coumaric acid, ferulic acid, and myristic acids, but a significant positive correlation with cinnamic acid. Available K exhibited significant negative correlations with p-coumaric acid and ferulic acids. Total P content was positively correlated with cinnamic acid, whereas total K was positively correlated with p-coumaric acid, ferulic, and myristic acids but a negative correlation with cinnamic acid. Overall, organic matter, available P, available K, total P, and total K content were identified as primary soil factors influencing allelochemical content in the soil, particularly p-coumaric acid, ferulic, myristic acids, and cinnamic acids. Overall, the analysis identified 11 significant positive and 6 significant negative correlations between agronomic traits and exudates, with positive correlations prevailing.

#### 3.8.2 Correlations between microbial genera and soil properties

**Figure 12:**
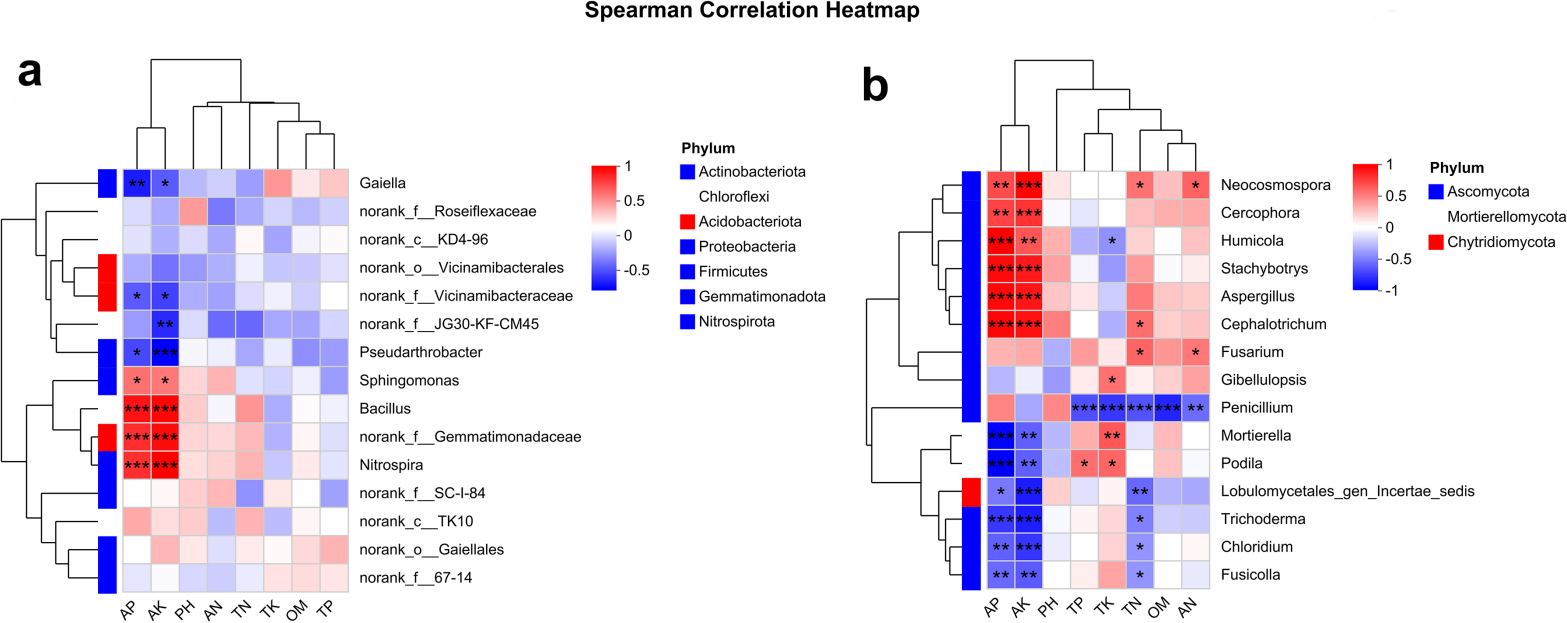
Correlation analysis of agrochemical properties and microbial bacteria **(a)** and fungi **(b)** in tobacco rhizosphere soil under different inoculation treatments. Red represents positive correlation; blue represents negative correlation. * Indicates a significant correlation between substances (*p< 0.05, **p< 0.01, ***p< 0.001).

Correlation analysis between the top 15 dominant bacterial genera and agronomic traits indicated that *Gaiella* and *Pseudomonas* were significantly negatively correlated with total P and K contents. In contrast, *Sphingomonas*, *Bacillus*, *Gemmatimonas*, and *Nitrospira* displayed significant positive correlations with these nutrients (**Figure 12a**). Among fungi, *Neocosmospora* exhibited significant positive correlations with total N, P, K, and alkaline N contents. *Trichoderma*, *Mortierella*, *Aspergillus*, and *Monodictys* also showed significant positive correlations with total P and K contents. *Fusarium* had significant positive correlations with total N and alkaline N contents, whereas *Penicillium* was significantly negatively correlated with total N, alkaline N, organic matter, available P, and available K contents. *Mortierella* showed significant negative correlations with total K and total P contents, but positive correlations with available K and available P contents. *Gemmatimonas*, *Trichoderma*, and *Mortierella* were significantly negatively associated with alkaline N, total P, and total K contents (**Figure 12b**). Overall, bacterial genera exhibited more negative correlations with agronomic traits, whereas fungal genera showed predominantly positive correlations.

#### 3.8.3 Partial least squares path modeling analysis of soil microbial diversity, soil nutrients, allelochemicals, and plant nutrients

**Figure 13:**
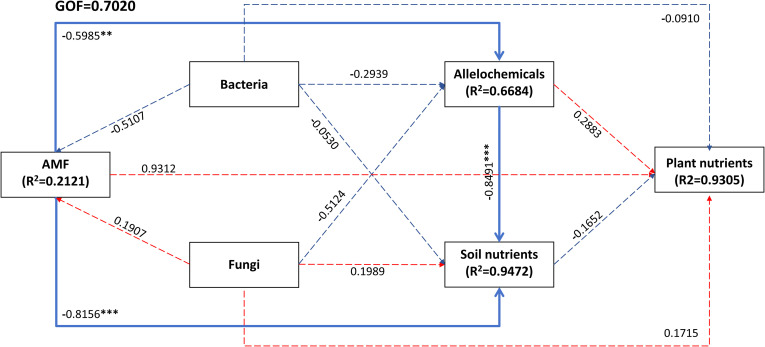
Partial least squares path modeling (PLS-PM) of soil microbial diversity, soil nutrients, allelochemicals and plant nutrients. Solid and dashed arrows indicate the significance of the pathway effects respectively. The numbers above the arrows represent the path coefficients. The model was evaluated using the goodness-of-fit (GOF = 0.7020). The R2 value represents the proportion of the variance of each variable that is explained.

This study employed Partial Least Squares Path Modeling (PLS-PM) to assess the interrelationships among soil microbial diversity, AMF colonization, allelochemicals, soil nutrients, and plant nutrients (**Figure 13**). The model’s Goodness-of-Fit was 0.7020, indicating a good model fit. The results revealed that soil bacteria were negatively correlated with AMF colonization, allelochemicals, soil nutrients, and plant nutrients; in contrast, soil fungi exhibited positive correlations with AMF colonization, soil nutrients, and plant nutrients, yet showed a negative correlation with allelochemicals. AMF colonization was positively correlated with plant nutrient content, while simultaneously demonstrating significant negative correlations with both soil nutrients and allelochemicals.

Collectively, these findings indicate a positive association between soil fungi and AMF colonization; furthermore, AMF colonization is negatively correlated with the accumulation of allelochemicals but positively correlated with plant nutrient uptake—thereby establishing AMF as a central regulator of rhizosphere interactions.

## 4 Discussion

### 4.1 AMF alleviates continuous cropping-induced growth suppression in tobacco

Tobacco is highly susceptible to continuous cropping obstacles, as long-term monocultures deteriorate soil properties and microbial communities, ultimately reducing yield and quality, while simultaneously increasing the incidence of pests and disease (Xi et al., 2019; Chen et al., 2020). Our results confirm that continuous cropping significantly suppressed photosynthetic capacity, root development, and biomass accumulation in tobacco. In contrast, inoculation with *Funneliformis mosseae* effectively counteracted these negative effects (**Figure 1**; **Supplementary Table 2**), consistent with the well-established role of AMF in enhancing plant performance and ecosystem function (Choi et al., 2018). Wang (Wang et al., 2011) reported that mycorrhizal tobacco seedlings exhibited improved growth, yield, premium tobacco proportion, and economic value. Our findings demonstrate that AMF inoculation enhances photosynthesis and also promotes plant growth and increase aboveground dry weight under both continuous and non-continuous cropping conditions. Similarly, Xi and He (Xi and He, 2024) discovered that AMF similarly enhanced key photosynthetic parameters in cut-flower roses. Consistent with previous work, AMF inoculation in this study promoted tobacco growth and biomass, Zhao (Zhao et al., 2019) reported a growth promotion effect consistently documented in AMF-inoculated tobacco. Importantly, AMF inoculation significantly improved root system architecture, as evidenced by increased total root length, surface area, and number of forks (**Supplementary Table 1**). Enhanced root proliferation is a key indicator of growth and developmental status (Liu and Yuan, 2020), and this corroborates previous work demonstrating AMF enhanced root architecture in tobacco (Wu et al., 2012) Collectively, these results demonstrate that AMF inoculation systemically enhances photosynthesis, biomass production, and root development to counteract growth inhibition caused by continuous cropping.

### 4.2 AMF enhances the antioxidant defense system and reduces oxidative stress

Continuous cropping imposes oxidative stress on plants, as reflected by the elevated malondialdehyde (MDA) and proline (PRO) contents and reduced activities of antioxidant enzymes (SOD, POD, CAT) in both leaves and roots of tobacco (**Figure 3**; **Supplementary Tables 3**, **S4**). These observations align with studies showing long-term continuous cropping reduces plant resistance to environmental stressors (Yu et al., 2024), whereas AMF inoculation increase abiotic stress tolerance (Lian et al., 2019). Enzymes such as SOD, POD, and CAT are crucial for neutralizing reactive oxygen species (Song et al., 2014). In this study, the activities of SOD, CAT, and GSH in leaves and roots were higher in non-continuously cropped soils than in continuously cropped soils, likely because continuous cropping suppresses growth and decreases antioxidant activity (Zhou et al., 2023). Inoculation with AMF significantly increased the activities of SOD, POD, CAT, and PAL, as well as glutathione (GSH) content, while decreasing MDA and PRO levels (**Figure 3**). After AMF inoculation, antioxidant enzyme activities increased, indicating improved stress resistance (Liu et al., 2018). Astaneh et al. confirmed that PAL plays a critical role in plant responses to abiotic stress (Astaneh et al., 2018), the significant increase in PAL activity further indicates a shift toward enhanced synthesis of phenolic defense compounds. In addition, AMF inoculation increased GSH content in leaves and roots, consistent with findings by Xi (Xi and He, 2024), indicating that AMF broadly enhance antioxidant capacity in tobacco. A marked reduction in MDA following AMF inoculation, demonstrating effective containment of membrane lipid peroxidation (Lee et al., 2015; Lu et al., 2017), and MDA levels in leaves were higher in continuously cropped soils. Additionally, the observed significant decline in PRO content after AMF inoculation. Since PRO accumulation acts as a stress protectant (Li et al., 2024), it indicates a reduction in environmental stress (Tigka and Ipsilantis, 2020), is consistent with findings in AMF-associated crops of rice, that AMF inoculation increased crop yield (Norouzinia et al., 2020). In summary, AMF orchestrates a comprehensive antioxidant response that mitigates oxidative damage and enhances systemic stress resilience in tobacco facing continuous cropping obstacles.

### 4.3 AMF improves soil nutrient utilization and enzyme

Our baseline soil analysis (**Table 1**) revealed that continuous cropping soils actually accumulated significantly higher levels of total and available N, P, and K, likely due to historical over-fertilization by farmers attempting to counteract yield declines. Furthermore, the adjacent non-continuous cropping field (C-) had been conventionally cultivated with maize, which possesses significantly lower basal nutrient requirements and receives fewer top-dressings compared to tobacco, thereby explaining the observed baseline nutrient disparities. However, despite this nutrient abundance in the soil, the apparent nutrient use efficiency of tobacco was markedly reduced (**Table 3**). This indicates that the growth inhibition is driven by nutrient lock-up and autotoxicity rather than absolute nutrient depletion. AMF inoculation significantly improved plant N, P, and K accumulation (**Figure 2**) and increased their utilization rates, particularly under continuous cropping (**Table 3**). These findings are consistent with Guo (Guo et al., 2023) reported that AMF inoculation significantly increased N content in both leguminous and non-leguminous plants. Consistent with this, we found that AMF inoculation elevated N levels in tobacco. AMF facilitates host P uptake, thereby facilitating plant growth (Li et al., 2016). In this study, total soil P content declined after AMF inoculation, while plant P levels increased, likely because AMF enhanced soil P activation and subsequent absorption (Schroeder et al., 2013). Similarly, AMF inoculation also significantly increased root K content, which is consistent with the findings in goji berries that demonstrated greater K uptake following AMF inoculation (Zhang et al., 2017). Moreover, AMF improved N, P, and K utilization rates in tobacco, aligning with previous findings that AMF enhances these nutrient absorption efficiencies (Chandrasekaran, 2020). Continuous cropping soils markedly reduced their utilization compared to non-continuous cropping soils. This decline is likely attributable to nutrient imbalances in organic matter, available P, and available K contents that develop during prolonged continuous cropping (He et al., 2008).

Soil enzymes are critical mediators of in nutrient cycling. CAT functions as a key redox enzyme that mitigates harmful cellular effects (Yi et al., 2024). Inoculation with AMF and non-continuous cropping soils both significantly increased the activity of S-CAT. Feng (Feng and Chen, 2017) observed similar increases of S-CAT activity following AMF inoculation in kiwi. Soil ACP influences organic P decomposition and bioavailability and therefore serves as an indicator of soil available P (Wang et al., 2016). SC hydrolyzes sucrose into glucose and fructose, thereby affecting N, P, and K dynamics in soil (Li et al., 2017). In this study, continuous cropping significantly suppressed the activities of S-CAT, ACP, SC, and PPO (**Figure 4**; **Supplementary Table 6**), reflecting impaired biological activity and nutrient turnover. However, AMF inoculation enhanced these enzyme activities, including PPO. Liu (Liu et al., 2024) similarly reported substantial reductions in soil ACP activity under long-term continuous cropping compared with rotational systems. Hong et al. showed that continuous cropping of patchouli reduced CAT, SC, and phosphatase activities (Hong et al., 2020). AMF can elevate soil phosphatase and SC activities, supporting P activation and uptake as well as carbohydrate metabolism in plants (Ma, 2023). The stimulation of these enzymes indicates that AMF revitalizes soil biochemical functions, thereby improving nutrient availability and supporting plant growth in degraded continuous cropping soils.

### 4.4 AMF reshapes soil microbial communities in the tobacco rhizosphere

AMF inoculation exerted a stronger influence on the soil fungal community than on bacterial (**Figure 5**). Although AMF significantly increased bacterial α-diversity, it reduced the total number of bacterial and fungal OTUs, suggesting a selection for specific functional taxa. This pattern is consistent with observations in other crops, where AMF inoculation in watermelon markedly increased bacterial and actinomycete OTUs, whereas fungal abundance declined, consistent with observations in other crops that AMF altered microbial abundance (Xie et al., 2018). Across treatments, at the phylum level, Proteobacteria, Actinobacteria, Acidobacteria, Chloroflexota, and Gemmatimonadetes were the dominant bacterial phyla, and AMF increased the relative abundances of Proteobacteria, Acidobacteria, and Gemmatimonadetes (**Supplementary Figure 3**) – phyla associated with nutrient cycling, organic matter decomposition, and plant growth promotion. Ye (Ye et al., 2025) reported that AMF inoculation promoted actinomycete proliferation in soil. Gemmatimonadetes are oligotrophic bacteria associated with nutrient cycling and contribute to organic matter decomposition and N/P transformation (Zhang et al., 2019). *Acidobacteria* include acidophilic and oligotrophic chemolithoautotrophic taxa (Wang et al., 2016) involved in soil pH regulation and carbon cycling during humus decomposition (Chen et al., 2018). AMF inoculation increased the relative abundances of Gemmatimonadetes and Acidobacteria, thereby facilitating nutrient activation and utilization. At the genus level, AMF enriched beneficial taxa such as *Sphingomonas* and *Lysobacter* (**Figure 6c**), the latter being a well−known biocontrol agent. Concurrently, AMF suppressed potential pathogens including *Fusarium* and *Gibellulopsi*s (**Figure 6d**), indicating a shift toward a healthier rhizosphere microbiome.

Regarding fungal communities, Ascomycota, Zygomycota, Chytridiomycota, and Basidiomycota were the dominant phyla in tobacco rhizosphere soil. AMF increased the relative abundances of Ascomycota and Chytridiomycota (**Supplementary Figure 3**), which are involved in organic matter decomposition and complex carbon degradation, Zygomycota improve N utilization in tobacco and contribute to soil health (Tao, 2024). Basidiomycota decompose lignin and cellulose, enhancing nutrient cycling (Ma, 2020). Ascomycota include ectomycorrhizal fungi that establish mutualistic relationships with roots and play key roles in N and P cycling. Chytridiomycota decompose complex organic substrates such as cellulose and chitin (Helgason and Fitter, 2009). The results indicated that AMF inoculation significantly alters the soil fungal community composition, with a consistent trend toward increased relative abundances of Ascomycota and Chytridiomycota under different soil conditions. This shift promotes the cycling and availability of organic matter and essential nutrients, such as N and P (Liu et al., 2025). Conversely, continuous cropping soils inoculated with AMF typically exhibit reduced relative abundances of Basidiomycota, Zygomycota, and Zygomycetaceae, taxa whose overrepresentation may hinder efficient nutrient utilization by crops. Functional predictions using FUNGuild align with these taxonomic shifts, suggesting that AMF inoculation promotes a microenvironment that favors symbiotic fungi while potentially restricting the accumulation of taxa predicted to be pathogenic, a key mechanism in overcoming continuous cropping obstacles (Yu et al., 2022). Network analysis reveals that AMF inoculation under continuous cropping enhances complexity of microbial interaction (**Supplementary Table 7**), suggesting the development of a more stable, interconnected, and cooperative microbial network (Floc’h et al., 2022). Collectively, these shifts toward a microbiome with enhanced decomposing and nutrient-mobilizing capacities provide a mechanistic explanation for how AMF improves nutrient availability and alleviates the microbial dysfunction reduced by long-term continuous cropping.

### 4.5 AMF reduces accumulation of autotoxic allelochemicals

The accumulation of phytotoxic allelochemicals (autotoxins) in root exudates is a well-established mechanism driving continuous cropping obstacles, with compounds like benzoic and ferulic acids being major contributors to yield decline (Zhang et al., 2007; Wu, 2010; Gao et al., 2021). Liu (Liu and Tang, 2017) found that excessive p-hydroxybenzoic acid significantly reduced K content in tobacco leaves. Yang (Yang et al., 2020) documented increased vanillic and phenolic acids in soils under long-term continuous tobacco and Coptis cultivation (Liu et al., 2024). Additionally, continuous peanut cropping demonstrated that benzoic acid, cinnamic acid, p-hydroxybenzoic acid, and related allelochemicals substantially contribute to cropping obstacles and increase over time (Liu et al., 2015). In this study, continuous cropping significantly increased the contents of p-hydroxybenzoic acid, benzoic acid, cinnamic acid, and vanillic acid contents were higher in the rhizosphere soils, whereas it decreased the contents of coumaric acid, ferulic acid, and levels myristic acid (**Table 4**).This variation may reflect differences in allelochemical production and decomposition influenced by plant metabolism, microbial activity, and environmental conditions (Tharayil, 2009).

This study revealed that inoculation with arbuscular mycorrhizal fungi (AMF) resulted in a significant increase in the concentrations of benzoic acid and cinnamic acid (**Table 4**). Although these compounds are traditionally regarded as phytotoxic allelochemicals that exacerbate continuous cropping obstacles, their elevated levels within the AMF-treated rhizosphere suggest that they may play a more complex ecological role. We hypothesize that these substances do not merely function as autotoxins; rather, AMF may selectively stimulate the secretion of these specific phenolic acids, enabling them to act as “rhizosphere activators” or signaling molecules. Previous studies have demonstrated that cinnamic acid can serve as a chemoattractant for the genus Sphingomonas (Jia et al., 2025)—a bacterial group that, notably, exhibited significant enrichment in the AMF-treated group in this study. This differentiated regulatory mechanism underscores the fact that AMF do not indiscriminately suppress all root-derived phenolic secretions; instead, by selectively modulating the chemical composition of the rhizosphere, they construct beneficial microbial networks that ultimately serve to alleviate the challenges associated with continuous cropping.

Although this study did not directly conduct bioassays, existing literature allows us to infer the autotoxic potential of accumulated phenolic acids. For instance, previous studies on tobacco have confirmed that p-hydroxybenzoic acid—which significantly impairs antioxidant enzymes—severely disrupts the antioxidant enzyme system when its concentration exceeds 100 ng/g (Cheng et al., 2020). Similarly, vanillic acid—known for its potent autotoxic inhibitory effects in continuous cropping systems—has been shown to synergize with the proliferation of pathogenic microorganisms while inhibiting the colonization of beneficial microorganisms when its concentration exceeds 810 ng/g (Chen et al., 2021). Furthermore, other studies indicate that phenolic acids such as p-coumaric acid exert increasingly detrimental effects on tobacco growth as their concentrations rise; notably, the inoculation of AMF in the present study effectively reduced the concentrations of these phenolic acids (Jia et al., 2024).

### 4.6 Integrative analysis reveals AMF as a master regulator

The correlation analyses (**Figures 8**, **9**) and partial least squares path modeling (PLS-PM; **Figure 10**) provide a holistic view of the mechanisms by which AMF alleviates continuous cropping stress. Phenolic acids constitute the major components of tobacco root exudates (Zhang et al., 2013). In this study, coumaric acid and ferulic acid displayed significant negative correlations with rapid available K and available P content in the soil, consistent with findings by Lv (Lv et al., 2002), who found that total phenolic acids restrict the absorption of N, P, K, and other nutrients. Others, like cinnamic acid, may function as “rhizosphere activators,” potentially enhancing phosphorus availability through acidification (Mei et al., 2012). AMF colonization exerted negative effects on allelochemicals and enhanced bacterial and plant nutrient levels.

The PLS-PM model revealed that soil bacteria positively influenced AMF colonization, thereby enhancing plant nutrient content. Concurrently, AMF improved soil nutrient availability and suppressed allelochemical accumulation, while fungal communities contributed positively to soil and plant nutrient parameters. These findings support the hypothesis that AMF plays a central role in modulating the rhizosphere by suppressing inhibitors and leveraging beneficial microbes and nutrient-mobilizing processes. Specifically, AMF reduced the mobility of toxic allelochemicals through chelation and hyphal retention (Kaldorf et al., 1999). Hyphal surfaces can support bacteria capable of N fixation, P solubilization, and growth promotion, thereby strengthening root nutrient acquisition (Shi et al., 2021). These interactions ultimately facilitate nutrient uptake and accumulation in plants. This coordinated action—suppressing inhibitors while leveraging activators—highlights AMF as a key mediator that optimizes the rhizosphere for plant nutrient uptake.

Although this study provides comprehensive insights into the physiological and microbial mechanisms by which arbuscular mycorrhizal fungi (AMF) alleviate continuous cropping obstacles, the inherent limitations of pot experiments cannot be overlooked. The sterilized substrate mixtures and controlled greenhouse environments employed in these pot experiments are unable to fully replicate the complex physical structures, significant spatial heterogeneity, and intricate multi-trophic microbial interactions characteristic of natural field soils. Furthermore, the findings of this study are currently limited to a single crop growth cycle. Consequently, caution must be exercised when extrapolating these mechanisms to agricultural production systems at the field scale. Future research should prioritize conducting long-term, multi-year field trials across various soil types to validate the sustained effects and efficacy of AMF inoculants; concurrently, further in-depth investigations are required to determine precisely how complex field environmental variables influence the AMF-mediated restructuring of the rhizosphere and the detoxification of allelochemicals within tobacco continuous cropping systems. Secondly, this study utilized a single AMF strain (*Funneliformis mosseae*). Given that the efficacy of mycorrhizal symbiosis often exhibits high strain specificity, future research should evaluate the synergistic effects of multi-strain AMF consortia to develop more universally applicable bio-fertilizers for continuous cropping systems. Finally, the alleviation of autotoxicity observed in this study is primarily inferred from a quantitative reduction in soil allelochemicals. Future research must incorporate rigorously controlled gradient bioassays—such as seed germination and seedling physiological tests—to precisely determine the *in vivo* toxicity thresholds of individual and composite phenolic acids, thereby providing direct biological evidence for AMF-mediated detoxification.

## 5 Conclusion

This study demonstrates that inoculation with Funneliformis mosseae effectively alleviates continuous cropping obstacles in tobacco. Physiologically, AMF colonization significantly enhances host photosynthetic capacity and systemic antioxidant defense, mitigating oxidative damage. In the rhizosphere, AMF functions as a pivotal biological regulator, restoring soil homeostasis by increasing nutrient (N, P, K) bioavailability and essential soil enzyme activities. Crucially, AMF remodels the rhizosphere microenvironment by significantly reducing the accumulation of autotoxic allelochemicals and shifting the microbial community structure toward beneficial taxa (e.g., Sphingomonas) while suppressing potential pathogens. These integrated physiological and ecological mechanisms highlight the potential of AMF as a sustainable biotechnological approach to remediate soil degradation and improve crop productivity in intensive continuous cropping systems.

## Data Availability

The original contributions presented in the study are included in the article/**Supplementary Material**. Further inquiries can be directed to the corresponding authors.

## Supplementary material

Supplementary Figure 1 After 45 days of pot culture, the colonization morphology of AMF in the root cells of flue-cured tobacco inoculated with AMF. FIGURE a illustrates the arbuscular structure of AMF, figure b illustrates the structure of mycelium circle, figure c illustrates the structure of mycelium, and figure d illustrates the structure of vesicle.

Supplementary Figure 2 Growth and development of flue-cured tobacco plants after 45 days of different inoculation treatments.

Supplementary Figure 3 Community composition and abundance of bacteria **(a)** and fungi **(b)** at the phylum level in tobacco rhizosphere soil.

Supplementary Figure 4 LEfSe Multi-level Species Tree Diagram for Bacteria **(a)** and Fungi **(b)**.

Supplementary Figure 5 LDA Discriminant Bar Chart for Bacteria **(a)** and Fungi **(b)**

Supplementary Table 1 Total root length, total surface area, average diameter, total root volume, number of root tips and number of forks of flue-cured tobacco root after 45 days of different inoculation treatments (mean ± standard error, n = 3) * Indicates a significant difference between treatment groups under independent samples T-test (**p* < 0.05, ***p* < 0.01, ****p* < 0.001). In the two-factor analysis, * indicates a significant effect of the independent variable on the dependent variable under LSD, two-tailed test (NS: *p* > 0.05, **p<* 0.05, ***p* < 0.01, ****p* < 0.001). The asterisk on the right indicates the difference between treatment groups with and without AMF inoculation under the same soil conditions, and the asterisk on the left indicates the difference between continuous cropping soil and non-continuous cropping soil under the same inoculation conditions. Detailed Two-way ANOVA results, including F-values and effect sizes (η_p_^2^), are provided in **Supplementary Table 11**.

Supplementary Table 2 Effects of different inoculation treatments and soil types on tobacco growth (mean ± standard error, n = 3) * Indicates a significant difference between treatment groups under independent samples T-test (* *p* < 0.05, ** *p* < 0.01, *** *p* < 0.001). In the two-factor analysis, * indicates a significant effect of the independent variable on the dependent variable under LSD, two-tailed test (NS: *p* > 0.05, **p<* 0.05, ***p* < 0.01, ****p* < 0.001). The asterisk on the right indicates the difference between treatment groups with and without AMF inoculation under the same soil conditions, and the asterisk on the left indicates the difference between continuous cropping soil and non-continuous cropping soil under the same inoculation conditions. Detailed Two-way ANOVA results, including F-values and effect sizes (η_p_^2^), are provided in **Supplementary Table 12**.

Supplementary Table 3 Effects of arbuscular mycorrhizal fungi and continuous cropping soil on the antioxidant defense system of tobacco leaves. * Indicates a significant difference between treatment groups under independent samples T-test (* *p* < 0.05, ***p* < 0.01, ****p* < 0.001). In the two-factor analysis, * indicates a significant effect of the independent variable on the dependent variable under LSD, two-tailed test (NS: *p* > 0.05, **p<* 0.05, ***p* < 0.01, ****p* < 0.001). The asterisk on the right indicates the difference between treatment groups with and without AMF inoculation under the same soil conditions, and the asterisk on the left indicates the difference between continuous cropping soil and non-continuous cropping soil under the same inoculation conditions. Detailed Two-way ANOVA results, including F-values and effect sizes (η_p_^2^), are provided in **Supplementary Table 13**.

Supplementary Table 4 Effects of arbuscular mycorrhizal fungi and continuous cropping soil on the antioxidant defense system of tobacco roots. * Indicates a significant difference between treatment groups under independent samples T-test (**p* < 0.05, ***p* < 0.01, ****p* < 0.001). In the two-factor analysis, * indicates a significant effect of the independent variable on the dependent variable under LSD, two-tailed test (NS: *p* > 0.05, **p<* 0.05, ***p* < 0.01, ****p* < 0.001). The asterisk on the right indicates the difference between treatment groups with and without AMF inoculation under the same soil conditions, and the asterisk on the left indicates the difference between continuous cropping soil and non-continuous cropping soil under the same inoculation conditions. Detailed Two-way ANOVA results, including F-values and effect sizes (η_p_^2^), are provided in **Supplementary Table 14**.

Supplementary Table 5 Two-Factor Analysis Table of Soil Agro-chemical Properties * Indicates a significant difference between treatment groups under independent samples T-test (**p* < 0.05, ***p* < 0.01, ****p* < 0.001). In the two-factor analysis, * indicates a significant effect of the independent variable on the dependent variable under LSD, two-tailed test (NS: *p* > 0.05, **p<* 0.05, ***p* < 0.01, ****p* < 0.001). Detailed Two-way ANOVA results, including F-values and effect sizes (η_p_^2^), are provided in **Supplementary Table 15**.

Supplementary Table 6 Two-Factor Analysis Table of Soil Enzyme Activity * Indicates a significant difference between treatment groups under independent samples T-test (**p* < 0.05, ***p* < 0.01, ****p* < 0.001). In the two-factor analysis, * indicates a significant effect of the independent variable on the dependent variable under LSD, two-tailed test (NS: *p* > 0.05, **p<* 0.05, ***p* < 0.01, ****p* < 0.001). Detailed Two-way ANOVA results, including F-values and effect sizes (η_p_^2^), are provided in **Supplementary Table 16**.

Supplementary Table 7 Topological characteristics of the symbiotic network of soil bacterial and fungal genera

Supplementary Table 8 Two-Way ANOVA of the Effects of Continuous Cropping and AMF Inoculation on Arbuscular Mycorrhizal Fungal Colonization Rate (F-Values and Partial Eta-Squared)

Supplementary Table 9 Two-Way ANOVA of the Effects of Continuous Cropping and AMF Inoculation on N, P, and K Fertilizer Uptake Efficiency (F-Values and Partial Eta-Squared)

Supplementary Table 10 Two-Way ANOVA of the Effects of Continuous Cropping and AMF Inoculation on Phenolic Acid Accumulation in Tobacco Rhizosphere Soil (F-values and Partial Eta-squared)

Supplementary Table 11 Two-Way ANOVA of the Effects of Continuous Cropping and AMF Inoculation on Tobacco Rhizosphere Characteristics (F-Values and Partial Eta-Squared)

Supplementary Table 12 Two-Way ANOVA of the Effects of Continuous Cropping and AMF Inoculation on Tobacco Agronomic Traits (F-Values and Partial Eta-Squared)

Supplementary Table 13 Two-Way ANOVA of the Effects of Continuous Cropping and AMF Inoculation on Tobacco Leaf Enzyme Activity (F-Values and Partial Eta-Squared)

Supplementary Table 14 Two-Way ANOVA of the Effects of Continuous Cropping and AMF Inoculation on Tobacco Root Enzyme Activity (F-Values and Partial Eta-Squared)

Supplementary Table 15 Two-Way ANOVA of the Effects of Continuous Cropping and AMF Inoculation on Soil Nutrients (F-Values and Partial Eta-Squared)

Supplementary Table 16 Two-Way ANOVA of the Effects of Continuous Cropping and AMF Inoculation on Soil Enzyme Activity(F-Values and Partial Eta-Squared)
