# Supplementary material for: Effects of AMF on tobacco growth in continuous cropping soils: impacts on soil chemical properties and rhizosphere microbial diversity
Source: Front Plant Sci. 2026 Jul 8;17:1827990. doi: 10.3389/fpls.2026.1827990 (PMC13388823; doi:10.3389/fpls.2026.1827990)
Supplement: Supplementary Figure 1 — After 45 days of pot culture, the colonization morphology of AMF in the root cells of flue-cured tobacco inoculated with AMF. FIGURE a illustrates the arbuscular structure of AMF, figure b illustrates the structure of mycelium circle, figure c illustrates the structure of mycelium, and figure d illustrates the structure of vesicle. [file SupplementaryFile1.docx]

**Supporting materials**

**
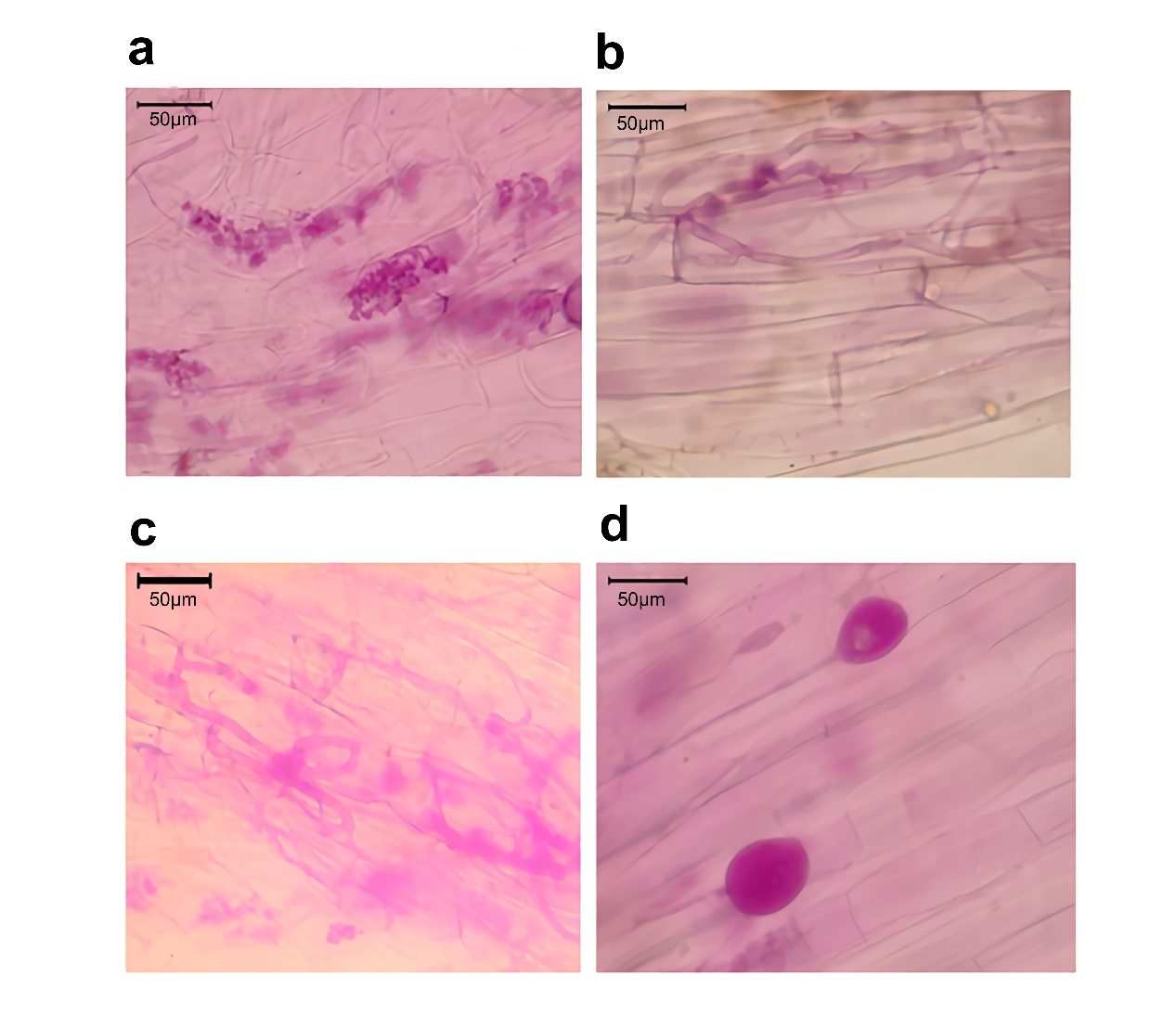
Figure S1.** After 45 days of pot culture, the colonization morphology of AMF in the root cells of flue-cured tobacco inoculated with AMF. FIGURE a illustrates the arbuscular structure of AMF, figure b illustrates the structure of mycelium circle, figure c illustrates the structure of mycelium, and figure d illustrates the structure of vesicle.


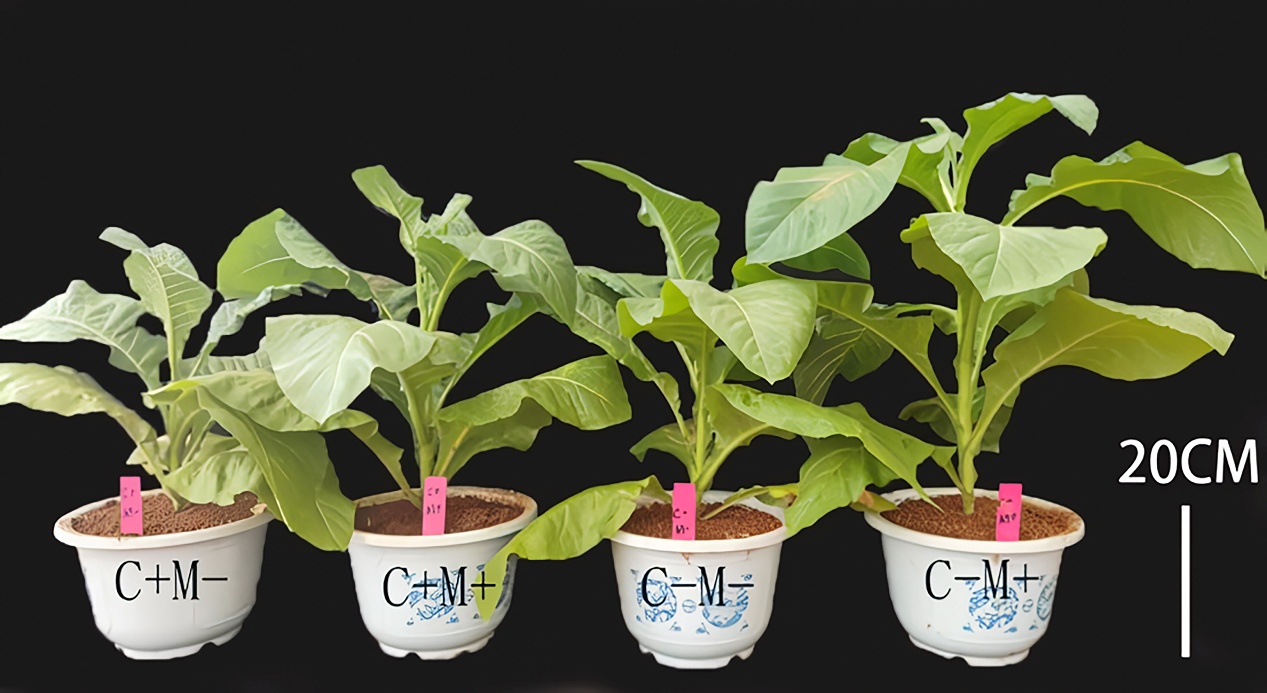


**
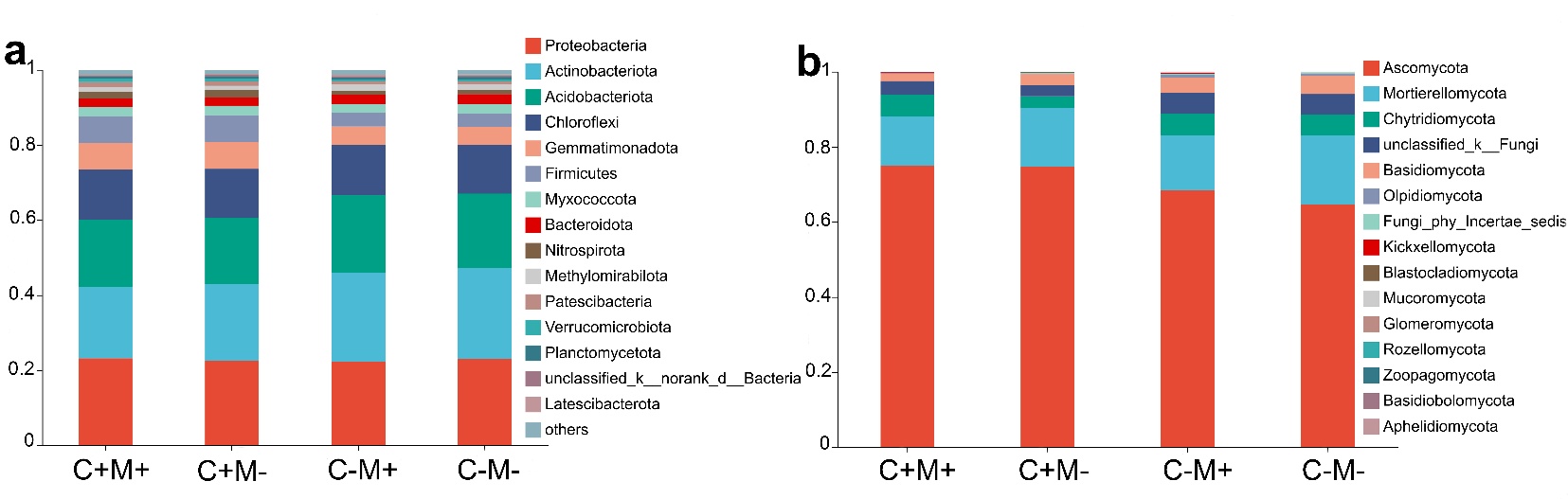
Figure S2.** Growth and development of flue-cured tobacco plants after 45 days of different inoculation treatments.

**Figure S3.** Community composition and abundance of bacteria (a) and fungi (b) at the phylum level in tobacco rhizosphere soil.


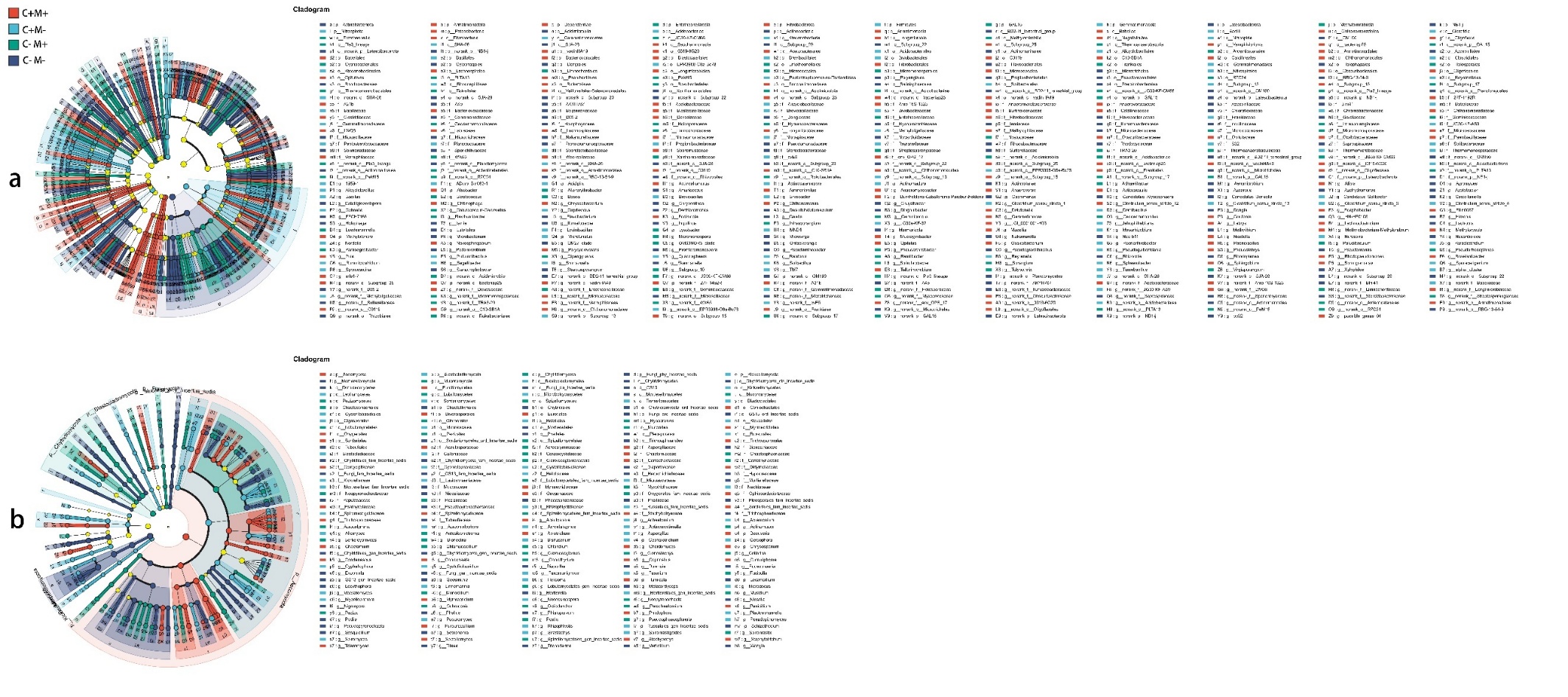
**Figure S4.** LEfSe Multi-level Species Tree Diagram for Bacteria (a) and Fungi (b).


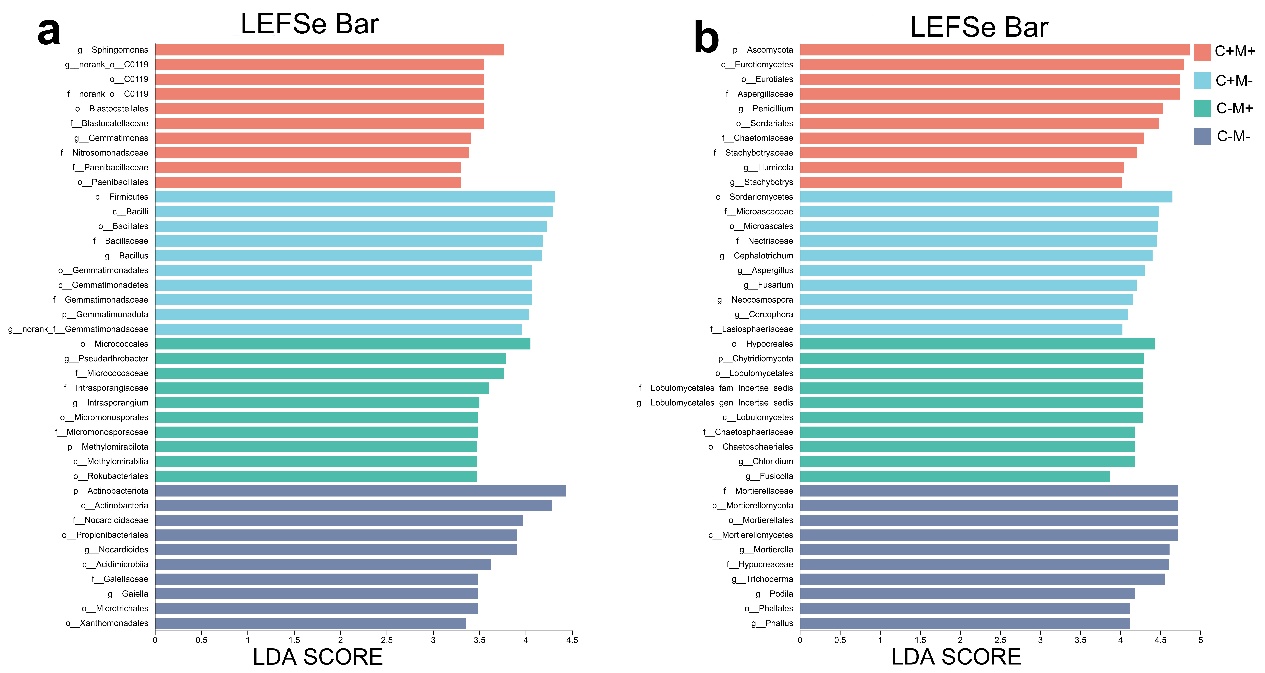


**Figure S5.** LDA Discriminant Bar Chart for Bacteria (a) and Fungi (b
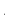
)

TableS1. Total root length, total surface area, average diameter, total root volume, number of root tips and number of forks of flue-cured tobacco root after 45 days of different inoculation treatments (mean ± standard error, n = 3)

| Treatments | | Length(cm) | Surf rea(cm^2^) | Avg Diam(mm) | Root Volume(cm^3^) | Tips | Forks |
| --- | --- | --- | --- | --- | --- | --- | --- |
| C+ | M- | 8314.36±519.78 | 839.53±90.06 | 0.3229±0.0109 | 6.7740±1.026 | 22087.33±2093.10 | 121596±1010.54 |
|  | M+ | 8982.12±74.55 | 962.84±71.14 | 0.3356±0.0002 | 8.0287±0.0769 | 22411±1338.39 | 131301.7±1029.58*** |
| C- | M- | *10106.29±96.23 | *1163.92±169.59 | *0.3748±0.0003 | *11.4467±0.1234 | 24512±1132.23 | 121951.3±1398.84 |
|  | M+ | ***10292.02±103.62 | 1176.92±162.57 | ***0.3786±0.0003*** | ***11.365±0.1326 | *25309±568.15 | ***149739.7±1499.46*** |
| Soil | P | *** | ** | *** | *** | * | *** |
| AMF | P | * | NS | * | NS | NS | *** |
| Soil*AMF | P | NS | NS | NS | NS | NS | *** |

* Indicates a significant difference between treatment groups under independent samples T-test (* *p* < 0.05, ** *p* < 0.01, *** *p* < 0.001). In the two-factor analysis, * indicates a significant effect of the independent variable on the dependent variable under LSD, two-tailed test (NS: *p* > 0.05, * *p* < 0.05, ** *p* < 0.01, *** *p* < 0.001). The asterisk on the right indicates the difference between treatment groups with and without AMF inoculation under the same soil conditions, and the asterisk on the left indicates the difference between continuous cropping soil and non-continuous cropping soil under the same inoculation conditions. Detailed Two-way ANOVA results, including F-values and effect sizes (η_p_^2^), are provided in Table S11.

TableS2.Effects of different inoculation treatments and soil types on tobacco growth (mean ± standard error, n = 3)

| Treatments | | Plant height  (cm) | Stem circumference  (cm) | Maximum leaf length  (cm) | Maximum leaf width  (cm) | Number of leaves | |
| --- | --- | --- | --- | --- | --- | --- | --- |
| C+ | M- | 40.94±2.16 | 4.08±0.26 | 44.08±1.08 | 21.6±0.82 | 10.0±0.00 | |
|  | M+ | 48.86±2.15*** | 4.34±0.27 | 45.36±0.96 | 22.24±0.57 | 11.2±0.45** | |
| C- | M- | ***47.56±1.43 | 4.3±0.35 | 42.26±3.51 | 21.36±1.85 | ***11.2±0.45 | |
|  | M+ | ***57.36±1.15*** | **4.96±0.26* | *49.36±2.37** | **24.34±0.72* | ***12.8±0.45*** | |
| Soil | P | *** | ** | NS | NS | *** | |
| AMF | P | *** | ** | ** | ** | *** | |
| Soil*AMF | P | NS | NS | * | * | | NS |

* Indicates a significant difference between treatment groups under independent samples T-test (* *p* < 0.05, ** *p* < 0.01, *** *p* < 0.001). In the two-factor analysis, * indicates a significant effect of the independent variable on the dependent variable under LSD, two-tailed test (NS: *p* > 0.05, * *p* < 0.05, ** *p* < 0.01, *** *p* < 0.001). The asterisk on the right indicates the difference between treatment groups with and without AMF inoculation under the same soil conditions, and the asterisk on the left indicates the difference between continuous cropping soil and non-continuous cropping soil under the same inoculation conditions. Detailed Two-way ANOVA results, including F-values and effect sizes (η_p_^2^), are provided in Table S12.

TableS3.Effects of arbuscular mycorrhizal fungi and continuous cropping soil on the antioxidant defense system of tobacco leaves.

| Treatments | | SOD  (u/g) | POD  (ΔOD470/min/g) | MDA  (nmol/g) | PRO  (ug/g) | CAT  (umol/min/g) | PAL  (ΔOD290/h/g) | GSH (umol/g) |
| --- | --- | --- | --- | --- | --- | --- | --- | --- |
| C+ | M- | 219.51±9.30 | 321.16±58.54 | 51.92±0.50** | 205.00±8.25** | 844.31±10.85 | 226.09±11.34 | 0.41±0.02 |
|  | M+ | 238.52±16.72 | 366.62±28.36 | 48.44±1.59 | 179.19±4.34 | 923.89±6.85*** | 478.84±46.82** | 0.52±0.06* |
| C- | M- | 228.58±3.16 | 313.94±16.41 | 50.35±0.58*** | 108.49±10.69*** | 1305.93±26.50 | 374.41±55.63 | 0.46±0.06 |
|  | M+ | 262.61±3.77*** | 678.68±40.20*** | 44.60±0.79 | 57.35±7.44 | 1540.47±15.66*** | 694.93±78.33** | 0.58±0.06 |
| M+ | C- | 262.61±3.77* | 678.68±40.20*** | 44.60±0.79 | 57.35±7.44 | 1540.47±15.66*** | 694.93±78.33* | 0.58±0.06 |
|  | C+ | 238.52±16.72 | 366.62±28.36 | 48.44±1.59** | 179.19±4.34*** | 923.89±6.85 | 478.84±46.82 | 0.52±0.06 |
| M- | C- | 228.58±3.16 | 313.94±16.41 | 50.35±0.58 | 108.49±10.69 | 1305.93±26.50*** | 374.41±55.63* | 0.46±0.06 |
|  | C+ | 219.51±9.30 | 321.16±58.54 | 51.92±0.50** | 205.00±8.25*** | 844.31±10.85 | 226.09±11.34 | 0.41±0.02 |
| Soil | P | ** | *** | *** | *** | *** | *** | NS |
| AMF | P | *** | *** | *** | *** | ** | *** | 5** |
| Soil*AMF | P | NS | *** | NS* | ** | *** | NS | NS |

* Indicates a significant difference between treatment groups under independent samples T-test (* *p* < 0.05, ** *p* < 0.01, *** *p* < 0.001). In the two-factor analysis, * indicates a significant effect of the independent variable on the dependent variable under LSD, two-tailed test (NS: *p* > 0.05, * *p* < 0.05, ** *p* < 0.01, *** *p* < 0.001). The asterisk on the right indicates the difference between treatment groups with and without AMF inoculation under the same soil conditions, and the asterisk on the left indicates the difference between continuous cropping soil and non-continuous cropping soil under the same inoculation conditions. Detailed Two-way ANOVA results, including F-values and effect sizes (η_p_^2^), are provided in Table S13.

TableS4.Effects of arbuscular mycorrhizal fungi and continuous cropping soil on the antioxidant defense system of tobacco roots.

| Treatments | | SOD  (u/g) | POD  (ΔOD470/min/g) | MDA (nmol/g) | PRO  (ug/g) | CAT (umol/min/g) | PAL  (ΔOD290/h/g) | GSH  (umol/g) |
| --- | --- | --- | --- | --- | --- | --- | --- | --- |
| C+ | M- | 212.24±9.55 | 1242.73±222.07 | 41.40±0.57*** | 43.16±4.69* | 405.54±2.58 | 103.88±2.55 | 0.4535±0.0716 |
|  | M+ | 195.30±14.72 | 1972.83±72.06** | 37.32±0.77 | 30.88±6.66 | 645.41±7.40*** | 209.98±4.41*** | 0.5727±0.0455 |
| C- | M- | 249.33±24.81 | 1293.97±172.76 | 44.08±3.07* | 13.23±0.94** | 1070.96±2.53 | 221.64±19.22 | 0.4903±0.03206 |
|  | M+ | 283.34±8.96* | 1354.80±123.66 | 39.56±0.39 | 9.33±2.28 | 1147.24±2.97*** | 405.52±22.75*** | 0.5965±0.0604 |
| M+ | C- | 283.34±8.96* | 1354.80±123.66 | 39.56±0.39** | 9.33±2.28 | 1147.24±2.97*** | 405.52±22.75** | 0.5965±0.0604 |
|  | C+ | 195.30±14.72 | 1972.83±72.06** | 37.32±0.77 | 30.88±6.66** | 645.41±7.40 | 209.98±4.41 | 0.5727±0.0455 |
| M- | C- | 249.33±24.81*** | 1293.97±172.76 | 44.08±3.07 | 13.23±0.94 | 1070.96±2.53*** | 221.64±19.22*** | 0.4903±0.03206 |
|  | C+ | 212.24±9.55 | 1242.73±222.07 | 41.40±0.57 | 43.16±4.69*** | 405.54±2.58 | 103.88±2.55 | 0.4535±0.0716 |
| Soil | P | *** | * | ** | *** | *** | *** | NS |
| AMF | P | NS | ** | *** | ** | *** | *** | ** |
| Soil*AMF | P | ** | ** | NS | * | *** | ** | NS |

* Indicates a significant difference between treatment groups under independent samples T-test (* *p* < 0.05, ** *p* < 0.01, *** *p* < 0.001). In the two-factor analysis, * indicates a significant effect of the independent variable on the dependent variable under LSD, two-tailed test (NS: *p* > 0.05, * *p* < 0.05, ** *p* < 0.01, *** *p* < 0.001). The asterisk on the right indicates the difference between treatment groups with and without AMF inoculation under the same soil conditions, and the asterisk on the left indicates the difference between continuous cropping soil and non-continuous cropping soil under the same inoculation conditions. Detailed Two-way ANOVA results, including F-values and effect sizes (η_p_^2^), are provided in Table S14.

TableS5. Two-Factor Analysis Table of Soil Agro-chemical Properties

| Treatments | pH | OM  (g/kg) | AN  (mg/kg) | AP  (mg/kg) | AK  (mg/kg) | TN  (g/kg) | TP  (g/kg) | TK  (g/kg) |
| --- | --- | --- | --- | --- | --- | --- | --- | --- |
| soil | NS | NS | NS | *** | *** | NS | NS | NS |
| AMF | NS | * | NS | *** | ** | ** | * | * |
| soil*AMF | NS | * | NS | ** | NS | * | NS | NS |

* Indicates a significant difference between treatment groups under independent samples T-test (* *p* < 0.05, ** *p* < 0.01, *** *p* < 0.001). In the two-factor analysis, * indicates a significant effect of the independent variable on the dependent variable under LSD, two-tailed test (NS: *p* > 0.05, * *p* < 0.05, ** *p* < 0.01, *** *p* < 0.001). Detailed Two-way ANOVA results, including F-values and effect sizes (η_p_^2^), are provided in Table S15.

TableS6 Two-Factor Analysis Table of Soil Enzyme Activity

| Treatments | S-CAT  (umol/h/g) | S-ACP  (umol/h/g) | S-SC  (mg/d/g) | PPO (nmol/h/g) |
| --- | --- | --- | --- | --- |
| Soil | *** | *** | *** | *** |
| AMF | *** | *** | *** | NS |
| soil*AMF | NS | *** | *** | NS |

* Indicates a significant difference between treatment groups under independent samples T-test (* *p* < 0.05, ** *p* < 0.01, *** *p* < 0.001). In the two-factor analysis, * indicates a significant effect of the independent variable on the dependent variable under LSD, two-tailed test (NS: *p* > 0.05, * *p* < 0.05, ** *p* < 0.01, *** *p* < 0.001). Detailed Two-way ANOVA results, including F-values and effect sizes (η_p_^2^), are provided in Table S16.

TableS7 Topological characteristics of the symbiotic network of soil bacterial and fungal genera

| Index | C+M- | C+M+ | C-M- | C-M+ |
| --- | --- | --- | --- | --- |
| Modularity (MD) | 0.451 | 0.367 | 0.343 | 0.606 |
| Average clustering coefficient | 0.533 | 0.55 | 0.559 | 0.546 |
| Average path length | 3.407 | 3.513 | 3.103 | 3.825 |
| Network diameter | 9 | 11 | 7 | 9 |
| Grath densitty | 0.09 | 0.133 | 0.131 | 0.077 |
| Average degree (AD) | 8.9 | 12.667 | 12.735 | 7.515 |
| Positive | 273 | 364 | 403 | 202 |
| Negative | 172 | 244 | 221 | 170 |
| Nodes | 100 | 96 | 98 | 99 |

TableS8 Two-Way ANOVA of the Effects of Continuous Cropping and AMF Inoculation on Arbuscular Mycorrhizal Fungal Colonization Rate (F-Values and Partial Eta-Squared)

| Two-way ANOVA |  | Arbuscule (%) | Hyphae (%) | Hyphal coil (%) | Vesicle (%) | Total (%) |
| --- | --- | --- | --- | --- | --- | --- |
| F | Soil | 78.102 | 6.511 | 1.946 | 15.158 | 7.253 |
|  | AMF | 9594.034 | 23.55 | 296 | 5.158 | 1428.113 |
|  | Soil* AMF | 48.949 | 6.055 | 31.135 | 3.789 | 4.717 |
| P | Soil | 0.000 | 0.021 | 0.182 | 0.001 | 0.016 |
|  | AMF | 0.000 | 0.000 | 0.000 | 0.037 | 0.000 |
|  | Soil* AMF | 0.000 | 0.026 | 0.000 | 0.069 | 0.045 |
| η_p_^2^ | Soil | 0.83 | 0.289 | 0.108 | 0.486 | 0.312 |
|  | AMF | 0.998 | 0.595 | 0.949 | 0.244 | 0.989 |
|  | Soil* AMF | 0.754 | 0.275 | 0.661 | 0.191 | 0.228 |

TableS9 Two-Way ANOVA of the Effects of Continuous Cropping and AMF Inoculation on N, P, and K Fertilizer Uptake Efficiency (F-Values and Partial Eta-Squared)

| Two-way ANOVA |  | N | P | K |
| --- | --- | --- | --- | --- |
| F | Soil | 177.597 | 58.742 | 52.076 |
|  | AMF | 53.426 | 207.392 | 8.603 |
|  | Soil* AMF | 23.608 | 19.883 | 1.895 |
| P | Soil | 0.000 | 0.000 | 0.000 |
|  | AMF | 0.000 | 0.000 | 0.019 |
|  | Soil* AMF | 0.001 | 0.002 | 0.206 |
| η_p_^2^ | Soil | 0.957 | 0.88 | 0.867 |
|  | AMF | 0.87 | 0.963 | 0.518 |
|  | Soil* AMF | 0.747 | 0.713 | 0.192 |

TableS10 Two-Way ANOVA of the Effects of Continuous Cropping and AMF Inoculation on Phenolic Acid Accumulation in Tobacco Rhizosphere Soil (F-values and Partial Eta-squared)

| Two-way ANOVA |  | P-hydroxybenzoic acid(ng/g) | Vanillic acid  (ng/g) | P-coumaric acid  (ng/g) | | Ferulic acid  (ng/g) | | Benzoic acid  (ng/g) | Cinnamic acid  (ng/g) | Myristic acid  (ng/g) |
| --- | --- | --- | --- | --- | --- | --- | --- | --- | --- | --- |
| F | Soil | 18.461 | 3.832 | 11206.998 | 458.562 | | 3.877 | | 4771.77 | 806.569 |
|  | AMF | 241.63 | 21.796 | 1865.338 | 95.602 | | 907.563 | | 785.795 | 609.75 |
|  | Soil* AMF | 6.329 | 3.08 | 181.465 | 6.185 | | 142.711 | | 504.315 | 394.338 |
| P | Soil | 0.001 | 0.074 | 0.000 | 0.000 | | 0.072 | | 0.000 | 0.000 |
|  | AMF | 0.000 | 0.001 | 0.000 | 0.000 | | 0.000 | | 0.000 | 0.000 |
|  | Soil* AMF | 0.027 | 0.105 | 0.000 | 0.029 | | 0.000 | | 0.000 | 0.000 |
| η_p_^2^ | Soil | 0.606 | 0.242 | 0.999 | 0.974 | | 0.244 | | 0.997 | 0.985 |
|  | AMF | 0.953 | 0.645 | 0.994 | 0.888 | | 0.987 | | 0.985 | 0.981 |
|  | Soil* AMF | 0.345 | 0.204 | 0.938 | 0.34 | | 0.922 | | 0.977 | 0.97 |

TableS11 Two-Way ANOVA of the Effects of Continuous Cropping and AMF Inoculation on Tobacco Rhizosphere Characteristics

(F-Values and Partial Eta-Squared)

| Two-way ANOVA |  | Length(cm) | Surf rea(cm^2^) | Avg Diam(mm) | Root Volume(cm^3^) | Tips | Forks |
| --- | --- | --- | --- | --- | --- | --- | --- |
| F | Soil | 97.606 | 14.327 | 227.414 | 176.294 | 10.93 | 168.551 |
|  | AMF | 7.39 | 0.735 | 6.906 | 3.782 | 0.484 | 670.883 |
|  | Soil* AMF | 2.357 | 0.461 | 1.945 | 4.908 | 0.086 | 156.044 |
| P | Soil | 0.000 | 0.005 | 0.000 | 0.000 | 0.011 | 0.000 |
|  | AMF | 0.026 | 0.416 | 0.03 | 0.088 | 0.506 | 0.000 |
|  | Soil* AMF | 0.163 | 0.516 | 0.201 | 0.058 | 0.776 | 0.000 |
| η_p_^2^ | Soil | 0.924 | 0.642 | 0.966 | 0.957 | 0.577 | 0.955 |
|  | AMF | 0.48 | 0.084 | 0.463 | 0.321 | 0.057 | 0.988 |
|  | Soil* AMF | 0.228 | 0.054 | 0.196 | 0.38 | 0.011 | 0.951 |

TableS12 Two-Way ANOVA of the Effects of Continuous Cropping and AMF Inoculation on Tobacco Agronomic Traits

(F-Values and Partial Eta-Squared)

| Two-way ANOVA |  | Plant height  (cm) | Stem circumference  (cm) | Maximum leaf length  (cm) | | Maximum leaf width  (cm) | Number of leaves |
| --- | --- | --- | --- | --- | --- | --- | --- |
| F | Soil | 90.24 | 10.595 | 1.185 | 3.499 | | 65.333 |
|  | AMF | 123.943 | 12.709 | 17.516 | 13.253 | | 65.333 |
|  | Soil* AMF | 1.395 | 2.402 | 8.449 | 5.538 | | 1.333 |
| P | Soil | 0.000 | 0.005 | 0.292 | 0.08 | | 0.000 |
|  | AMF | 0.000 | 0.003 | 0.001 | 0.002 | | 0.000 |
|  | Soil* AMF | 0.255 | 0.141 | 0.01 | 0.032 | | 0.265 |
| η_p_^2^ | Soil | 0.849 | 0.398 | 0.069 | 0.179 | | 0.803 |
|  | AMF | 0.886 | 0.443 | 0.523 | 0.453 | | 0.803 |
|  | Soil* AMF | 0.08 | 0.131 | 0.346 | 0.257 | | 0.077 |

TableS13 Two-Way ANOVA of the Effects of Continuous Cropping and AMF Inoculation on Tobacco Leaf Enzyme Activity

(F-Values and Partial Eta-Squared)

| Two-way ANOVA |  | SOD  (u/g) | POD  (ΔOD470/min/g) | MDA  (nmol/g) | PRO  (ug/g) | CAT (umol/min/g) | | PAL  (ΔOD290/h/g) | GSH (umol/g) |
| --- | --- | --- | --- | --- | --- | --- | --- | --- | --- |
| F | Soil | 14.092 | 75.964 | 39.015 | 929.341 | | 41932.068 | 34.49 | 3.445 |
|  | AMF | 36.046 | 137.549 | 114.245 | 115.444 | | 3559.067 | 85.359 | 14.859 |
|  | Soil* AMF | 2.891 | 83.332 | 6.884 | 12.501 | | 866.127 | 1.193 | 0.005 |
| P | Soil | 0.002 | 0.000 | 0.000 | 0.000 | | 0.000 | 0.000 | 0.101 |
|  | AMF | 0.000 | 0.000 | 0.000 | 0.000 | | 0.000 | 0.000 | 0.005 |
|  | Soil* AMF | 0.108 | 0.000 | 0.018 | 0.003 | | 0.000 | 0.307 | 0.945 |
| η_p_^2^ | Soil | 0.468 | 0.826 | 0.709 | 0.983 | | 1 | 0.812 | 0.301 |
|  | AMF | 0.693 | 0.896 | 0.877 | 0.878 | | 0.998 | 0.914 | 0.65 |
|  | Soil* AMF | 0.153 | 0.839 | 0.301 | 0.439 | | 0.991 | 0.13 | 0.001 |

TableS14 Two-Way ANOVA of the Effects of Continuous Cropping and AMF Inoculation on Tobacco Root Enzyme Activity

(F-Values and Partial Eta-Squared)

| Two-way ANOVA |  | SOD  (u/g) | POD  (ΔOD470/min/g) | MDA  (nmol/g) | PRO  (ug/g) | CAT (umol/min/g) | | PAL  (ΔOD290/h/g) | GSH (umol/g) |
| --- | --- | --- | --- | --- | --- | --- | --- | --- | --- |
| F | Soil | 77.99 | 9.673 | 11.105 | 182.627 | | 53319.346 | 322.613 | 0.93 |
|  | AMF | 1.452 | 18.835 | 36.153 | 18.054 | | 3911.352 | 276.353 | 12.83 |
|  | Soil* AMF | 12.933 | 13.486 | 0.054 | 4.83 | | 1047.354 | 19.878 | 0.043 |
| P | Soil | 0.000 | 0.014 | 0.004 | 0.000 | | 0.000 | 0.000 | 0.363 |
|  | AMF | 0.246 | 0.002 | 0.000 | 0.001 | | 0.000 | 0.000 | 0.007 |
|  | Soil* AMF | 0.002 | 0.006 | 0.819 | 0.043 | | 0.000 | 0.002 | 0.841 |
| η_p_^2^ | Soil | 0.83 | 0.547 | 0.41 | 0.919 | | 1 | 0.976 | 0.104 |
|  | AMF | 0.083 | 0.702 | 0.693 | 0.53 | | 0.998 | 0.972 | 0.616 |
|  | Soil* AMF | 0.447 | 0.628 | 0.003 | 0.232 | | 0.992 | 0.713 | 0.005 |

TableS15 Two-Way ANOVA of the Effects of Continuous Cropping and AMF Inoculation on Soil Nutrients

(F-Values and Partial Eta-Squared)

| Two-way ANOVA |  | pH | OM  (g/kg) | AN  (mg/kg) | AP  (mg/kg) | AK  (mg/kg) | | TN  (g/kg) | TP  (g/kg) | TK  (g/kg) |
| --- | --- | --- | --- | --- | --- | --- | --- | --- | --- | --- |
| F | Soil | 0.335 | 0.113 | 0.202 | 129.168 | | 72.075 | 5.127 | 0.312 | 4.484 |
|  | AMF | 1.874 | 9.245 | 0.073 | 35.505 | | 15.44 | 16.813 | 6.736 | 8.124 |
|  | Soil* AMF | 0.898 | 7.107 | 3.232 | 14.281 | | 0.237 | 5.697 | 1.105 | 1.918 |
| P | Soil | 0.578 | 0.746 | 0.665 | 0.000 | | 0.000 | 0.053 | 0.592 | 0.067 |
|  | AMF | 0.208 | 0.016 | 0.794 | 0.000 | | 0.004 | 0.003 | 0.032 | 0.021 |
|  | Soil* AMF | 0.371 | 0.029 | 0.11 | 0.005 | | 0.64 | 0.044 | 0.324 | 0.203 |
| η_p_^2^ | Soil | 0.04 | 0.014 | 0.025 | 0.942 | | 0.9 | 0.391 | 0.038 | 0.359 |
|  | AMF | 0.19 | 0.536 | 0.009 | 0.816 | | 0.659 | 0.678 | 0.457 | 0.504 |
|  | Soil* AMF | 0.101 | 0.47 | 0.288 | 0.641 | | 0.029 | 0.416 | 0.121 | 0.193 |

TableS16 Two-Way ANOVA of the Effects of Continuous Cropping and AMF Inoculation on Soil Enzyme Activity(F-Values and Partial Eta-Squared)

| Two-way ANOVA |  | S-CAT  (umol/h/g) | S-ACP  (umol/h/g) | S-SC  (mg/d/g) | PPO (nmol/h/g) |
| --- | --- | --- | --- | --- | --- |
| F | Soil | 42.298 | 150.521 | 4701.742 | 366.885 |
|  | AMF | 102.699 | 253.923 | 3532.281 | 0.025 |
|  | Soil* AMF | 1.499 | 128.979 | 57.009 | 1.035 |
| P | Soil | 0.000 | 0.000 | 0.000 | 0.000 |
|  | AMF | 0.000 | 0.000 | 0.000 | 0.877 |
|  | Soil* AMF | 0.239 | 0.000 | 0.000 | 0.339 |
| η_p_^2^ | Soil | 0.726 | 0.95 | 0.998 | 0.979 |
|  | AMF | 0.865 | 0.969 | 0.998 | 0.003 |
|  | Soil* AMF | 0.086 | 0.942 | 0.877 | 0.115 |
